# Impact of childhood adversity on acute subjective effects of stimulant and opioid drugs: Evidence from placebo-controlled studies in healthy volunteers

**DOI:** 10.1177/02698811241268892

**Published:** 2024-08-08

**Authors:** Molly Carlyle, Harriet de Wit, Siri Leknes

**Affiliations:** 1 Department of Psychology, University of Oslo, Blindern, Oslo, Norway; 2 Department of Psychology, University of Queensland, St Lucia, QLD, Australia; 3 Department of Psychiatry and Behavioral Neuroscience, University of Chicago, Chicago, IL, USA; 4 Department of Diagnostic Physics, Oslo University Hospital, Oslo, Norway

**Keywords:** Adversity, trauma, childhood, reward, amphetamine, methamphetamine, buprenorphine, opioid, subjective

## Abstract

**Background and Aims:**

Early-life adversities are known to alter drug reward processing in rodents. Despite the well-known link between early adversity and the risk of substance use disorder, few studies have measured how childhood adversity affects human drug reward. Here, we assessed the relationship between historical childhood adversities and responses to single doses of methamphetamine, d-amphetamine or buprenorphine in healthy participants.

**Methods:**

Using a secondary analysis approach, we assessed the impact of childhood adversity on drug effects from three randomised, placebo-controlled studies in which healthy volunteers received methamphetamine (20 mg oral; *n* = 35), d-amphetamine (20 mg oral; *n* = 54) or buprenorphine (0.2 mg sublingual; *n* = 35). Ratings of *feeling effect, liking, disliking, feeling high* and *wanting more* of the drug were collected 15–210 min post-administration, and heart rate changes were analysed using random-intercept mixed-effect models. The area under the curve from these and previous studies was calculated to visualise the relationship between childhood adversity severity and drug effects.

**Results:**

Greater childhood adversity was associated with reduced *feel effects* (significant three-way interactions *b* = −0.07, 95% CI [−0.12, −0.02], *p* = 0.009), *like effects* (*b* = −0.07, 95% CI [−0.13, −0.00], *p* = 0.038) and *feel high* (*b* = −0.06, 95% CI [−0.10, −0.01], *p* = 0.020) towards the stimulant drugs 90–180 min post-administration.

**Conclusions:**

Childhood adversity was not significantly associated with other subjective or heart rate responses to the drugs. Overall, participants with more childhood adversities reported dampened subjective responses to stimulant drugs, but not to buprenorphine. Future studies should examine the generalisability of these relationships, to identify the mechanisms underlying the link between childhood adversity and drug responsiveness.

## Introduction

Childhood experiences of abuse and neglect are highly prevalent among people with substance use problems (Santo et al., 2021) and are a significant risk factor that precedes and predicts use (Santo et al., 2022). While many factors likely contribute to this link, one potentially important mechanism may be a heightened positive subjective response to psychoactive drugs in people with childhood adversities (Oltean et al., 2023). Positive subjective responses to drugs predict abuse liability (Comer et al., 2012), and thus understanding the individual risk factors associated with positive drug responses is important for prevention and early interventions for addiction.

Rodent models of early life adversity have typically used early maternal deprivation and limited bedding and nesting as models of disrupted maternal care. The adult offspring were shown to exhibit greater self-administration of substances and stronger conditioned place preference (Vazquez et al., 2005), as well as slower extinction for drug-paired place preference and quicker reinstatement of drug-seeking behaviours (Levis et al., 2022). These behavioural indices of increased drug reward after early life adversities have been demonstrated across a range of substances, including opioids in male (Vazquez et al., 2005) and female rats (Levis et al., 2021), alcohol in male mice (Portero-Tresserra et al., 2018), cocaine in male (but not female) rats (Ganguly et al., 2019) and sucrose in male rats (Michaels and Holtzman, 2006). Another study found that early life adversity had the greatest effects on morphine reward, with some effect on amphetamine reward, but no effect on the consumption of alcohol or cocaine (Vazquez et al., 2006). Other studies have reported null effects, however. Male rats with early adversity did not show an enhanced preference for methamphetamine (Faure et al., 2009), cocaine (Bolton et al., 2018) or increased sucrose consumption (Shalev and Kafkafi, 2002). Some of the divergent effects of adversity are related to sex, where early adversity is thought to affect females and males differently during development (Rincón-Cortés, 2023). In sum, the preclinical literature indicates a causal relationship between early life adversities on drug reward that depends on neurocircuitry and sex.

Fewer studies have probed the effects of early adversity on the rewarding effects of drugs in humans after drug administration. One randomised placebo-controlled study selectively recruited healthy participants that either reported severe or no childhood adversity history (Carlyle et al., 2021). The subgroup with childhood adversity reported more subjective euphoria, liking and desire to receive more drugs after morphine administration (0.15 mg/kg intramuscularly). They also reported lower adverse effects such as dislike, nausea and dizziness compared to participants with no adversity history. These findings mirrored the preclinical data on enhanced drug reward after early adversity. However, the findings were not replicated in a follow-up study that probed the linear relationship between childhood adversity scores and drug liking of open-label pre-surgical opioids (Carlyle et al., 2023). Another human study administered the stimulant amphetamine (0.3 mg/kg intravenously) to healthy participants with varying levels of childhood adversities (Oswald et al., 2014). While subjective effects were not the primary objective of this study, the authors reported a significant interaction effect between the number of childhood adversities and gender on the pleasurable subjective effects of the drug. Post hoc analyses of this interaction indicated a non-significant trend to suggest that male participants with more adversities expressed more positive drug effects, while females reported the opposite. Although the sample size was small (*n* = 28) and levels of childhood adversity were low, these findings are in line with the preclinical findings of divergent sex effects. Finally, one other randomised, placebo-controlled study examined the subjectively aversive effects of oral delta-9-tetrahydrocannabinol in people on methadone treatment for opioid use disorder (Rogan et al., 2024). This brief exploratory analysis reported reduced aversive effects among people with high levels of childhood adversities.

Early life stress is thought to lead to disruptions in reward processing through enduring developmental changes in neurocircuitry related to stress and reward processing, which are not yet fully understood (see Levis et al., 2022, for a review). Preclinical studies indicate that early life adversity changes dopamine and opioid signalling, particularly in reward circuitry (Levis et al., 2022). Few studies have examined the neurobiological basis of early adversity and reward processing in humans. A positron-emission tomography (PET) study found that individuals with severe adversity exhibited lower striatal dopamine functioning than participants with no history of childhood adversity (Bloomfield et al., 2019). One study in healthy adults (with low levels of adversity) found that greater childhood adversity was related to greater amphetamine-induced dopamine release in the ventral striatum as measured by PET (Oswald et al., 2014). To our knowledge, there are no PET studies on opioid signalling in humans after childhood adversity, making it difficult to translate the preclinical insights to humans. Two studies that examined insecure attachment styles (a proxy of early life challenges) reported lower opioid receptor availability compared with people with secure attachment types (Nummenmaa et al., 2015; Turtonen et al., 2021).

In the current study, we probed the impact of early life adversities on drug reward using data from double-blind, randomised placebo-controlled studies in which healthy adults received single doses of the psychoactive drugs methamphetamine, d-amphetamine (both dopamine agonists) and buprenorphine (a partial opioid agonist) (Bershad et al., 2018; Murray et al., 2021; Van Hedger et al., 2018). The present analysis was designed to assess whether childhood adversity scores were linearly associated with acute drug reward, as measured by greater ratings of *liking, wanting* and *feeling high* and lower ratings of *disliking*. We hypothesised that participants with more childhood adversities would report greater *liking, wanting* and *feel high* and less *dislike* after the stimulant and opioid drugs – consistent with the majority of preclinical findings and small number of human findings (Carlyle et al., 2021; Ganguly et al., 2019; Levis et al., 2021, 2022; Michaels and Holtzman, 2006; Oswald et al., 2014; Portero-Tresserra et al., 2018; Rogan et al., 2024; Vazquez et al., 2005, 2006). Secondary exploratory analyses then assessed the link between childhood adversities and physiological drug responses (heart rate changes). These analyses were conducted to shed light on whether any observed differences in subjective drug responses were underscored by biological differences e.g, in pharmacokinetics.

Another secondary exploratory aim was to visualise the assumed cumulative (linear) relationship between childhood adversities and subjective responses – including comparison with other available human trial data using morphine (Carlyle et al., 2021), and pre-surgical oxycodone and remifentanil (Carlyle et al., 2023). This visual exploration was to facilitate comparison between the studies while also examining the linear impacts of adversity (assumed by the majority of the literature; Hamby et al., 2021), or the possible curvilinear effects (reported in some studies e.g., on stress resilience and mental health (Shapero et al., 2015)). Together, the insights from this research will build on the small number of human research studies and bridge preclinical findings on adversity-related changes to drug responses. The between-study comparison will attempt to synthesise and provide a more nuanced perspective on the inconsistencies in the literature by exploring the drug-dependent differences alongside ‘dose-dependent’ effects of childhood adversity.

## Methods

### Participants and measurement of childhood adversity

This was a secondary analysis of data collected by the *Human Behavioural Psychopharmacology Laboratory* at the *University of Chicago*. Adults from a community sample aged 18–40 were recruited to participate in studies involving the administration of a drug. Participants underwent an in-person medical screening that involved a physical, psychiatric and historical substance use assessment, to exclude individuals with likely DSM-5 Axis 1 disorders, substance use problems, physical health problems, contraindicating medications, pregnancy or lactation and BMI below 18 or above 30 (more information on exclusion and exclusion criteria are available in the original articles; Bershad et al., 2018; Murray et al., 2021; Van Hedger et al., 2018). All three studies were performed in accordance with the Declaration of Helsinki and approved by the University of Chicago’s institutional review board.

Experiences of childhood adversity were measured during screening, where participants completed the Childhood Trauma Questionnaire (CTQ) – a 25-item measure with five subscales including emotional, physical and sexual abuse, and emotional and physical neglect (Bernstein et al., 1997). Participants were asked to rate the frequency of these different types of traumatic experiences in childhood on a 5-point Likert scale (1 – none of the time, 5 – all of the time). A total CTQ score was calculated by summing responses across all 25 items, with the potential range of 25–125, while individual subscales scores ranged from 5 to 25. Predefined categories for none, low, moderate and severe childhood adversity were determined for each subscale (Bernstein et al., 1997; see Supplemental Material (SM) 1 for the established cut-off scores).

We included data from three studies conducted from 2011 to 2017. The studies used within-subjects designs that were placebo-controlled, double-blinded with randomised drug order. In the three studies, participants received methamphetamine, d-amphetamine and buprenorphine.

### Acute drug administration studies

#### Study 1

In this study (Van Hedger et al., 2018), 61 healthy adults received oral methamphetamine (20 mg) and placebo during four sessions (two with methamphetamine and two with placebo). Subjective drug responses and physiological measures were measured before and 15, 30, 70, 115 and 200 minutes after administration of the capsules. The study was designed to examine the effect of methamphetamine on conditioning and its corresponding neural correlates.

#### Study 2

In this study (Murray et al., 2021), 112 healthy adults received oral d-amphetamine (20 mg) and placebo during four sessions (two with d-amphetamine and two with placebo). Subjective and physiological responses were measured before and 30, 60, 90, 120, 150, 180 and 200 minutes post-drug. The study was designed to examine predictors of drug choice.

As methamphetamine and d-amphetamine are stimulants with very similar subjective and behavioural effects (Martin et al., 1971), data from Studies 1 and 2 were pooled for the current analyses. Preliminary tests confirmed the subjective response profiles between the two drugs did not significantly differ between these studies prior to pooling (SM2). Both studies involved two drug and placebo sessions with equivalent doses and identical procedures, as necessary for the original study’s purpose; however, the current analysis was only concerned with the overall drug and placebo effect.

#### Study 3

In this study (Bershad et al., 2018), 38 participants who reported a range of symptoms of depression and anxiety received sublingual buprenorphine (0.2 mg) and placebo across two sessions. Subjective and physiological responses were measured before and 30, 60, 180 and 210 minutes post-drug. The primary goal of this study was to examine the role of buprenorphine in emotional processing.

### Measurement of subjective and cardiovascular drug effects

In all three studies, subjective responses to the drug were assessed using the Drug Effects Questionnaire (DEQ; Morean et al., 2013). Using a 100-point visual analogue scale (0 – not at all, 100 – extremely), this measure asked participants to rate: (1) Do you FEEL a drug effect right now? (2) Are you HIGH right now? (3) Do you LIKE any of the effects you are feeling right now? (4) Do you DISLIKE any of the effects you are feeling right now? and (5) Would you like MORE of the drug you took, right now? Each time the DEQ was collected, physiological responses were also collected by measuring heart rate and blood pressure.

### Statistical analysis

All data were analysed using R v4.1.1. To examine the effect of childhood adversity on subjective responses to the drugs (stimulants and buprenorphine), mixed-effect random intercept models were developed to estimate a CTQ × Drug × Time interaction on *feel effects, like effects, dislike effects, feel high* and *want more*, measured by the DEQ. All models were fit by maximum likelihood estimation and included ID and session as random intercepts (ID/session). Fixed effects of CTQ scores and Time were entered as continuous variables, Drug (drug or placebo) was entered as categorical variable. Models adjusted for age and sex, in addition to Study for the stimulant analysis, by adding these as regressors in the models. Session was included as random intercept to control for between-session variation in subjective responses across the identical drug/placebo sessions in each study, while Study was included as a fixed effect to control for between-study variation. Timepoints for the stimulant analyses were coded at 0 (pre-drug), 15, 30, 65, 90, 117, 150, 180 and 205 min, where 0, 30, 65, 117 and 205 min were either exact or temporally close (⩽10 min) between Studies 1 and 2. Timepoints 15, 90, 150 and 180 min were not possible to match across the two studies (Study 1 did not have timepoints 90, 150 or 180, while Study 2 did not have timepoint 15), and therefore were left as missing values for the corresponding study. To assess the impact of the missing data and ensure the reliability of the results, two sensitivity analyses were conducted that (1) excluded these timepoints entirely from the analyses or (2) linearly imputed these timepoints with mean of the subjective responses before and after (i.e. using a moving average). Timepoints for the buprenorphine analysis were at 0, 30, 60, 180 and 210 min.

Estimates, confidence intervals and *p*-values are reported. For any significant interactions between childhood adversity and time, the models were re-run with time entered as a factor variable, and baseline as the contrast time point, to identify the exact timepoints for the significant differences. Alpha level was <0.05. The same analyses were also run on physiological heart rate (beats per minute) responses to the drug that were collected at the identical timepoints.

The normality of residuals was assessed for both random and fixed effects, in addition to heteroscedasticity for each predictor and homogeneity of variance using Levene’s test. Visual inspection of these was conducted using histograms and Q-Q plots. To address deviations with normality, all models were followed with Bootstrapping using random sampling with replacement (1000 iterations) using the BootMer package, which specified that we wanted to extract the fixed effects estimates and include random effects in the bootstrapping procedure. Bootstrapping as a method of addressing non-normality provides several advantages, including preserving the original data values and the parametric analysis strategy, and increasing robustness by enabling comparison of the original and bootstrapped estimates and 95% confidence intervals (reported in the Supplemental Material).

Exploratory analyses to visually examine the relationships between CTQ and overall drug responses were conducted using scatterplots. Overall drug responses were converted to total area under the curve (AUC) using the trapezoid rule across the drug and placebo sessions separately. Each was converted to a percentage AUC (scale 0–100) to assist comparison across studies where the duration and measurement times varied. The placebo AUC was subtracted from the drug AUC for a placebo-adjusted drug AUC. To comprehensively compare the relationships between CTQ and subjective responses across study drugs, we also included newly calculated AUC data from the previously published study data with morphine responses (Carlyle et al., 2021), and with a pre-surgical opioid (Carlyle et al., 2023) (the study latter was a single response, non-placebo controlled study, and so responses were converted to a 0–100 scale instead of AUC). Inclusion of the previously published data was done to explore any reliability of patterns in drug responses, to ease comparison across all the available data and to explore any similarities or inconsistencies in patterns of responding due to drug type, study design or CTQ severity.

## Results

### Summary descriptives and childhood adversity across the studies

CTQ responses from the screening were available for 35 (57%) participants from Study 1, 54 (48%) participants from Study 2 and 35 (92%) participants from Study 3. Possible reasons for missing CTQ responses for Studies 1 and 2 include the following: having completed the screening outside of the date range extracted here, or voluntary incompletion of the CTQ (which was not necessary for participation in the studies). Of these 124 participants, the average age was 24 years (Table 1). Approximately half of the participants were male for the stimulant studies, while there were slightly more women in the buprenorphine study (63%). Childhood adversity scores were generally low (average total scores between 32.5 and 38.5, on a scale ranging between 25 and 125), as expected for a non-clinical sample that was selected on an absence of psychiatric problems.

**Table 1.** Sample descriptives across the three studies using methamphetamine (*n* = 35), d-amphetamine (*n* = 54) and buprenorphine (*n* = 35).

		Stimulants (*n* = 89)		Buprenorphine (*n* = 35)
		Methamphetamine (*n* = 35)	d-amphetamine (*n* = 54)	
Age	*M* (SD)	23.7 (5.2)	24.2 (2.5)	23.5 (5)
	Range	18–35	21–29	18–40
Sex (*n* female)	*n*, %	18, 51.4%	25, 46.3%	22, 62.9%
BMI	*M* (SD)	22.9 (1.8)	22.9 (1.9)	23.98 (3.2)
Illicit drug use (ever used)				
Cannabis	*n*, %	30, 86%	44, 81%	24, 68%
Stimulants	*n*, %	16, 46%	13, 24%	9, 28%
Opioids	*n*, %	11, 31%	6, 11%	3, 9%
Hallucinogens	*n*, %	17, 49%	17, 32%	13, 37%
Tranquillisers	*n*, %	9, 26%	3, 6%	4, 11%
MDMA	*n*, %	13, 37%	9, 17%	5, 14%
Childhood adversity – averages				
Total score (range: 25–125)	*M* (SD)	38.5 (15)	32.5 (7.6)	36.9 (10.8)
	Range	25–84	25–61	25–70
Emotional abuse (5–25)	*M* (SD)	9.1 (4.9)	7.2 (2.5)	8.7 (4)
	Range	5–22	5–15	5–19
Emotional neglect (5–25)	*M* (SD)	9.8 (5.4)	7.6 (3.3)	9.6 (4.5)
	Range	5–23	5–17	5–19
Physical abuse (5–25)	*M* (SD)	7.2 (3.3)	6.1 (1.9)	6 (1.6)
	Range	5–18	5–15	5–11
Physical neglect (5–25)	*M* (SD)	7.2 (3.3)	6.1 (1.9)	6 (1.6)
	Range	5–18	5–15	5–11
Sexual abuse (5–25)	*M* (SD)	5.8 (2.5)	5.5 (2.5)	6.1 (3.5)
	Range	5–17	5–23	5–18
Childhood adversity – categories				
‘None’ on all subscales	*n*, %	11, 31%	31, 57%	13, 37%
‘Low-moderate’ on ⩾1 subscale	*n*, %	15, 43%	20, 37%	14, 40%
‘Severe’ on ⩾1 subscale	*n*, %	7, 20%	2, 4%	7, 20%
Emotional abuse	*n*, %	4, 11%	0, 0%	2, 6%
Emotional neglect	*n*, %	4, 11%	0, 0%	4, 11%
Physical abuse	*n*, %	1, 3%	0, 0%	1, 3%
Physical neglect	*n*, %	2, 6%	1, 2%	0, 0%
Sexual abuse	*n*, %	2, 6%	1, 2%	3, 9%
Drug effects (percentage AUC, 0–100)				
Drug feel	*M* (SD)	25.1 (16.8)	31.4 (17.9)	25.2 (15.9)
	Range	0.1–61.2	0.1–71.5	0–59.2
Placebo feel	*M* (SD)	10 (11.3)	10.1 (13.5)	14.3 (15.5)
	Range	0.1–33.8	0–57.2	0–58.1
Drug liking	*M* (SD)	43.3 (22.6)	44.2 (21.4)	27.7 (18.1)
	Range	0.9–87.3	0–75	0–59.8
Placebo liking	*M* (SD)	14.3 (18.6)	12.2 (15.4)	27.6 (23.5)
	Range	0–79.5	0–49.4	0.1–74
Drug disliking	*M* (SD)	13.8 (13.1)	15.1 (12.1)	29.5 (20.6)
	Range	0–43.4	0–45.7	0–74.4
Placebo disliking	*M* (SD)	14.5 (17.6)	15.9 (20.2)	22.8 (24)
	Range	0–74	0–66.3	0–83.4
Drug high	*M* (SD)	20.6 (17.2)	25.0 (19.1)	13.1 (12.5)
	Range	0–63.2	0–63.8	0–12.5
Placebo high	*M* (SD)	6 (9.4)	6.9 (11.8)	7.6 (10.7)
	Range	0–32.9	0–57.1	0–40
Drug more	*M* (SD)	45.8 (26.6)	40.4 (24.8)	21 (16.6)
	Range	0–84.6	0–79.1	0–54.1
Placebo more	*M* (SD)	12.4 (17.7)	11.2 (16.8)	20.1 (24.8)
	Range	0–66.7	0–73.5	0–79.6

Percentage AUC is calculated to provide an overall estimate of the drug effect across the session over all timepoints, and the percentage is used to allow comparison across each study. CTQ total scores range from 25 to 125, with the lowest scores of 25. Categories for childhood adversity could not be determined for *n* = 2 in Study 1, *n* = 1 in Study 2 and *n* = 1 in Study 3 due to missing data on one or more items of a subscale. AUC: area under the curve; CTQ: Childhood Trauma Questionnaire.

### Effect of childhood adversity on stimulant responses

**Figure 1.**
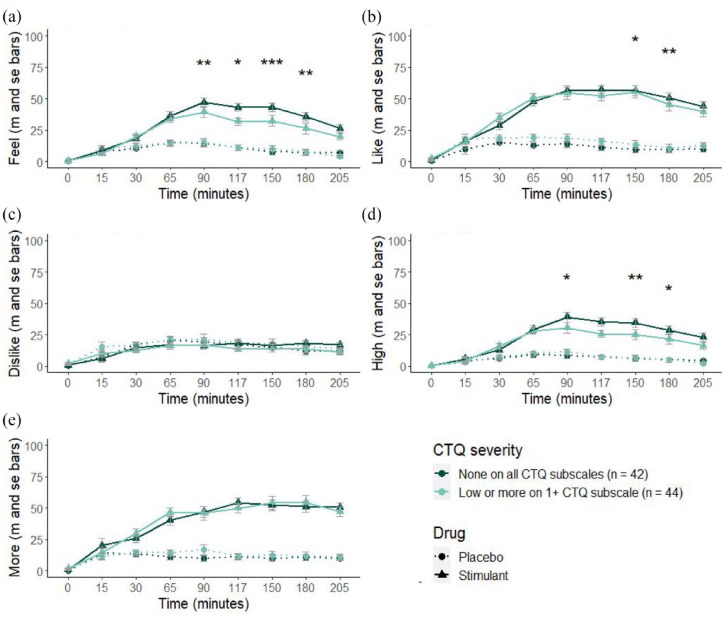
Subjective responses of (a) *feel effects*, (b) *like effects*, (c) *dislike effects*, (d) *feel high* and (e) want more to the stimulant and placebo doses between baseline and 205 min post-administration. Clear Drug × Time effects were observed for ratings of *feel effect, liking, feeling high* and want more, but not for drug disliking. Childhood adversity as measured by Childhood Trauma Questionnaire (CTQ) scores were treated as a continuous variable in all analyses; significant Drug × Time × CTQ score interactions are marked with asterisks. For illustration purposes, we group participants into those with no childhood trauma (scoring ‘none’ across all subscales) and those with low, moderate or severe childhood trauma on ⩾1 subscale. Response scores were averaged across the two administrations of stimulant and placebo. *p < 0.05, **p < 0.01, ***p < 0.001.

Participants who scored higher on the CTQ reported lower subjective *feel effects, like effects and feel high* after the stimulant drug over time (respectively: *b* = −0.07, 95% CI [−0.12, −0.02], *p* = 0.009; *b* = −0.07, 95% CI [−0.13, −0.00], *p* = 0.038; *b* = −0.06, 95% CI [−0.10, −0.01], *p* = 0.020); all estimates from maximum-likelihood based random intercept models for the significant CTQ × Drug × Time interactions are reported in SM3). As illustrated in Figure 1, this lower response to the stimulant drug was observed as a smaller change from baseline for *feel effects* at 90–180 min, *like effects* at 150–180 min and *feel high* at 90, 150 and 180 min post-administration (estimates for each time point are reported in SM3 – Table 2) (the linear effect of childhood adversity is not apparent in the illustration of *like effects* in Figure 1(b) where subgroup means are displayed; see Figure 4(b) for scatterplots). There were no significant effects of childhood adversity on *dislike effects* (*b* = −0.03, 95% CI [−0.09, 0.02], *p* = 0.220) or *want more* (*b* = −0.07, 95% CI [−0.10, 0.03], *p* = 0.249). There were significant Drug × Time interactions where participants reported greater *feel effects, like effects, feel high* and *want more* after the stimulant drug compared with placebo. There was no such effect for *dislike effects* between the placebo and stimulant sessions, and no significant effects of age, gender or study on any subjective outcome (SM3 Table 1).

Results from the two sensitivity analyses that either (1) excluded the timepoints that were not possible to match between Study 1 and 2 (15, 90, 150 and 180 min post-drug) or (2) linearly imputed the missing values for these time points, are reported in SM5. When only timepoints present in both studies were included, the three-way interaction effect of CTQ score × Drug × Time on *feel effects, like effects* and *feel high* was no longer significant (SM5 – Table 1). When linear imputation was employed to account for missing data from Study 2 at these timepoints, the interactions remained significant yet with a lower effect size (as measured by estimates; SM5 – Table 2), indicating consistency in effect.

Exploratory sensitivity analyses on significant effects were also conducted separately for males and females to examine potential sex effects on the relationship between childhood adversity and stimulant drug effects. We found the strongest effects in women, but no evidence of opposing patterns between men and women (models reported in SM6).

### Effect of childhood adversity on buprenorphine responses

**Figure 2.**
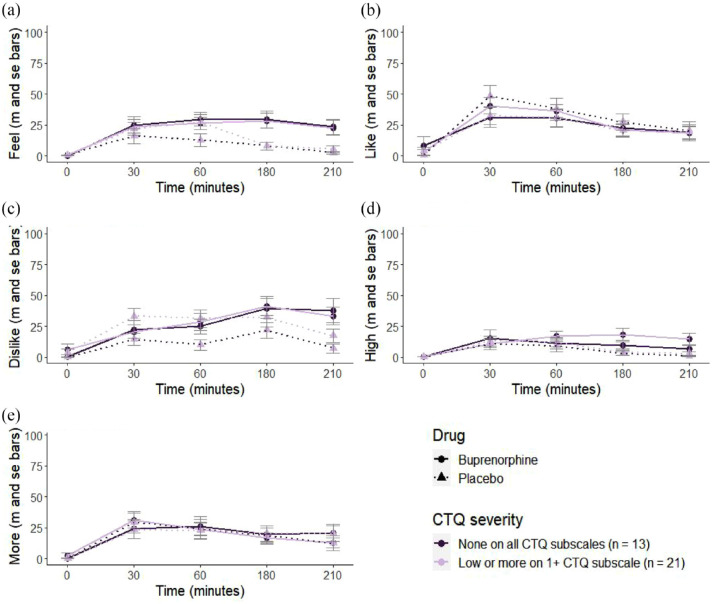
Subjective responses of (a) *feel effects*, (b) *like effects*, (c) *dislike effects*, (d) *feel high* and (e) want more to buprenorphine and placebo doses between baseline and 210 min post-administration. Responses to buprenorphine were not clearly different from placebo for any of the drug effects. We found no significant interactions between drug, time and Childhood Trauma Questionnaire (CTQ) scores. CTQ was treated as a continuous variable in all analyses. For illustration purposes, we group participants into those with no childhood trauma (scoring ‘none’ across all subscales) and those with low, moderate or severe childhood trauma on >1 subscale).

The effects of the buprenorphine dose were overall modest and did not differ from placebo. There was a main effect of Time for *feel effect, dislike* and *high*, showing that responses increased throughout the drug and placebo sessions (*feel effect: b* = 12.12, 95% CI [4.83, 19.40], *p* = 0.001; *dislike b* = 13.50, 95% CI [4.43, 22.58], *p* = 0.004; *high b* = 4.89, 95% CI [0.12, 9.66], *p* = 0.044). There were no significant effects of Drug or Time for *like effects* or *want more* (SM4 – Table 1). There were no significant effects of CTQ score on drug responses to buprenorphine compared with placebo over time (as indicated by no significant CTQ × Drug × Time interactions; maximum-likelihood-based random intercept models controlling for age and sex are reported in SM2 – Table 1). We did find support that higher CTQ scores were associated with reduced *feel effects* overall across time and drug condition (*b* = −0.19, 95% CI [−0.38, −0.00], *p* = .045; Figure 2); however, further inspection of this two-way interaction between CTQ × Time at each timepoint yielded non-significant results (SM4 – Table 2). There were no other significant interaction effects with CTQ and no significant effects of age or sex (SM4 – Table 1).

### Effect of drug on heart rate responses

**Figure 3.**
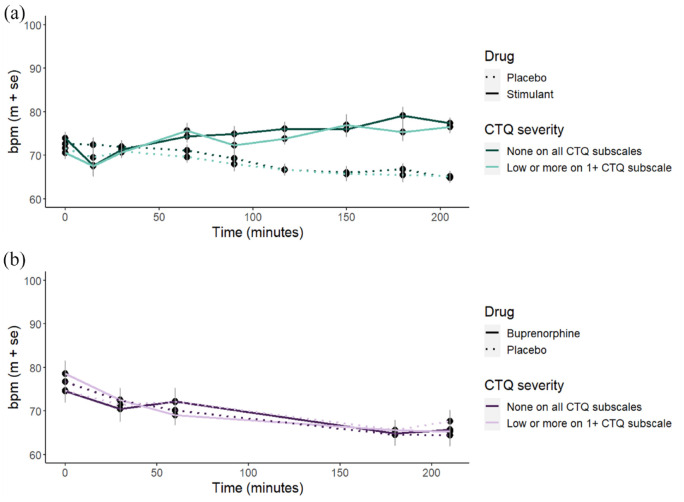
Average heart rate (bpm) after (a) stimulants and (b) buprenorphine. While stimulants significantly increased heart rate, there were no significant effects for buprenorphine or interactions with CTQ scores. For illustration purposes, we group participants into those with no childhood trauma (scoring ‘none’ across all subscales) and those with low, moderate or severe childhood trauma on >1 subscale). bpm: beats per minute; CTQ: Childhood Trauma Questionnaire.

The stimulant drugs increased heart rate (*b* = 0.07, 95% CI [0.04, 0.11], *p* < 0.001), whereas buprenorphine did not (*b* = 0.01, 95% CI [−0.05, 0.08], *p* = 0.692). Childhood adversity was not related to changes in heart rate after either stimulants or buprenorphine (Figure 3; all model outcomes reported in SM9).

### Visualisation of the linear effect of childhood adversity on drug responses

To visually inspect the relationship between childhood adversity scores and drug responses, we generated one drug effects score per participant by calculating the AUC for drug versus placebo. Each AUC value was then displayed against CTQ scores using scatterplots for each rating and each study: responses to methamphetamine (Study 1), d-amphetamine (Study 2) and buprenorphine (Study 3). For completeness, we also computed AUC data from previously published studies: responses to intramuscular morphine (Carlyle et al., 2021), and the average (non-placebo controlled) drug responses to pre-surgical opioids (remifentanil or oxycodone) delivered on the operating table (Carlyle et al., 2023). All studies included assessed childhood adversity using the CTQ.

**Figure 4.**
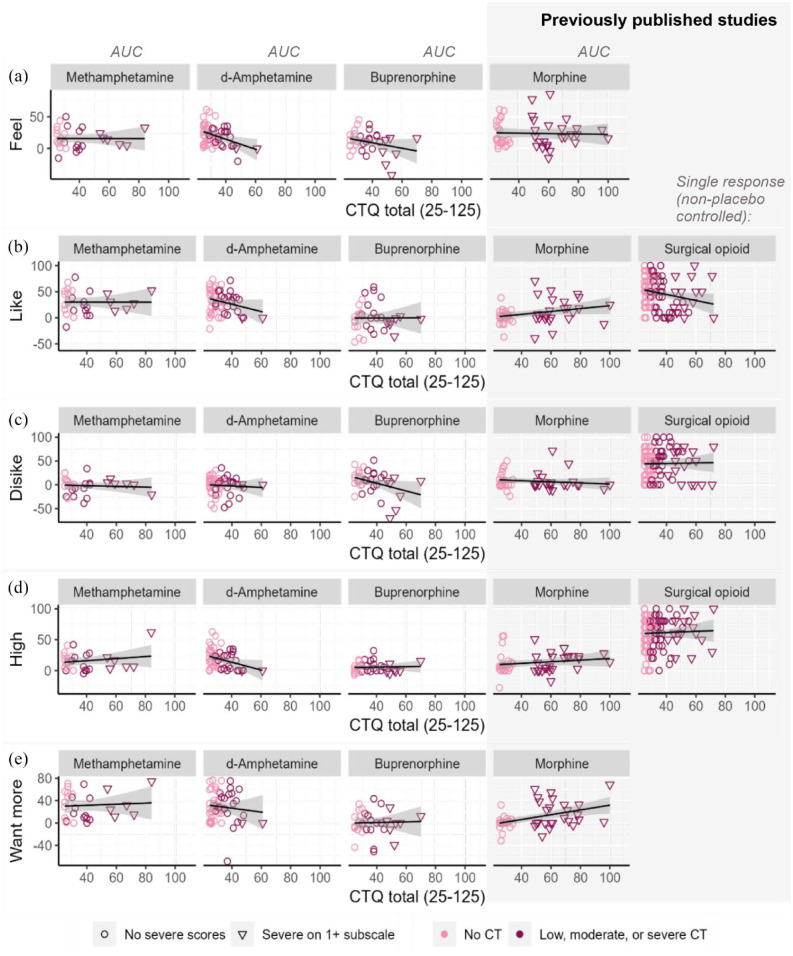
Visualisation of the linear relationship between childhood trauma and drug responses for (a) *feel effects*, (b) *like effects*, (c) *dislike effects*, (d) *feel high* and (e) want more using total area under the curve (AUC) controlling for placebo after methamphetamine, d-amphetamine, buprenorphine or morphine and non-placebo controlled response (0–100) after a surgical opioid (remifentanil or oxycodone). The data for morphine and surgical opioids are from two previously published studies. The surgical opioid study was an observational, non-placebo controlled study that collected responses for *liking, disliking* and *high* only once, and therefore AUC could not be calculated. AUC values for all the experimental, placebo-controlled studies were calculated (drug AUC-placebo AUC) and converted to percentages to allow for comparison across the studies with different measurement times.

As illustrated in Figure 4, the study samples varied in the range of CTQ scores included, with maximum scores of 60 for the d-amphetamine study and scores up to 100 in the previously published morphine study. Overall, there was not a clear, consistent linear relationship between childhood adversity across drugs or subjective effect; however, several negative associations were apparent for d-amphetamine, buprenorphine and surgical opioids. By contrast, morphine responses tended to increase with higher CTQ scores, although there was no apparent linear relationship within the CTQ group for most measures. The small yet significant negative linear effect for stimulant liking reported in the section ‘Summary descriptives and childhood adversity across the studies’, Figure 1, can be observed in Figure 4. Examination of Figure 4 shows that Study 1 with d-amphetamine likely drove the significant interactions with CTQ observed in the primary analyses with stimulants. The pattern for methamphetamine is less conclusive but importantly does not provide support for an opposite effect. Another figure illustrating the linear associations between childhood adversity scores and drug responses via subgroups is included in SM9, which indicates a consistent direction of effect between the two stimulant studies. We include an illustration of the pattern of results separately for women and men in SM11.

## Discussion

This study sought to identify whether childhood adversities were associated with the altered affective experience of psychoactive drugs. We tested this in a secondary analysis of responses to stimulants (methamphetamine and d-amphetamine) and one opioid drug (buprenorphine) in healthy adults with varying levels of childhood adversity. Participants received single doses of these psychoactive drugs under double-blind conditions. We found that greater childhood adversity was associated with dampened responses to the stimulant effects, specifically for feeling the effects, liking the effects and feeling high. Although the samples included few people with high childhood adversities, the present findings indicate that even low levels of childhood adversities may have enduring impacts on the subjective experience of stimulant drugs.

We found no evidence that childhood adversity affected stimulants’ effect on heart rate, nor ratings of disliking or wanting more of these medications. Likewise, we found no clear indication that responses to buprenorphine were altered by childhood adversities. For stimulant disliking and all ratings of buprenorphine, responses were low overall and did not significantly differ from placebo, which could be due to limited statistical power and low drug dosage. Unlike previous studies (e.g., Ganguly et al., 2019; Oswald et al., 2014), we did not find support for any effects of sex on subjective drug responses in the primary analyses; however, exploratory analyses indicated that the reduced stimulant feeling, liking and high may be driven by females with higher childhood adversity. Descriptive exploration of the linear effects of childhood adversity using AUC between the current study and prior research studies did not indicate a consistent linear relationship between childhood adversity and subjective drug effects; however, negative associations appeared more often than positive associations.

The finding of dampened subjective responses to stimulants is partly consistent with prior research that shows altered acute responses to drugs in people with childhood adversities (Carlyle et al., 2021, 2023; Oswald et al., 2014), however, the direction and specific effects differ: Prior studies reported increased pleasurable effects of morphine (Carlyle et al., 2021), decreased liking of surgical opioids (Carlyle et al., 2023) and increased liking of amphetamine (in men only; Oswald et al., 2014) in people with more childhood adversities. The inconsistent association between childhood adversity and drug reward is mirrored in the animal literature, where behavioural paradigms assess drug consumption and motivation to obtain more of the drug. Some studies reported increased drug reward to opioids (Levis et al., 2021, 2022; Vazquez et al., 2005, 2006), alcohol (Portero-Tresserra et al., 2018), cocaine (Ganguly et al., 2019), amphetamines (Vazquez et al., 2006) and sucrose (Michaels and Holtzman, 2006) in rodents exposed to early life adversities. On the contrary, other studies report no effects for alcohol (Vazquez et al., 2006), cocaine (Bolton et al., 2018), methamphetamine (Faure et al., 2009) or sucrose consumption (Shalev and Kafkafi, 2002). The results share some similarities with blunted subjective feelings of alcohol effects among young men fathered by alcoholics (Schuckit, 1984); however, this finding has not been consistently replicated (Morzorati et al., 2002; Söderpalm Gordh and Söderpalm, 2011). While parts of the human and animal literature support an effect of early life stress on the drug experience, the divergent or lack of effects across drug types warrants further investigation if we are to harness this knowledge for clinical use.

The negative association between CTQ scores and ratings of stimulant drug liking reported here could support drug-dependent differences in subjective reward after childhood adversity. One preclinical study reported a specific susceptibility to opioid reward over other drug types (such as stimulants) after early life adversity (Vazquez et al., 2006). However, the rodents exposed to adversity still showed higher reward responses to amphetamines than controls, inconsistent with our human stimulant findings. Furthermore, the reduced subjective liking of the stimulant may simply be a result of less feeling of effect, and we cannot conclude with certainty that people with more childhood adversities would experience less subjective reward at larger doses. Differences in dose and routes of administration may also contribute to inconsistencies, where the drugs were administered orally in this study compared with weight-adjusted intramuscular (Carlyle et al., 2021) or intravenous (Carlyle et al., 2023; Oswald et al., 2014) administration procedures. While the subjective liking of drug effects is associated with consumption in humans (Oltean et al., 2023), behavioural reward paradigms, such as effort-based self-administration tasks or patient-controlled analgesia pumps, would provide a more nuanced perspective on these findings, and draw closer parallels with preclinical rodent findings.

Participants reported feeling the effects of buprenorphine. However, unlike prior studies with other opioids such as morphine (Carlyle et al., 2021) or remifentanil and oxycodone (Carlyle et al., 2023), there were no significant interactions in drug responses related to childhood adversity. For many participants, the drug responses were very low and did not exceed placebo, and low doses are not typically reported as pleasurable or euphoric in healthy volunteers (Bershad et al., 2016, 2018). However, greater positive effects have been reported at higher doses in clinical groups (Huhn et al., 2019; Pathak et al., 2019), where the impacts of childhood adversity may be observable. Given the pharmacological differences between morphine (a full mu-opioid receptor agonist) and buprenorphine (a partial mu-opioid receptor agonist and kappa receptor antagonist), exploration of the impacts of childhood adversity and greater buprenorphine doses could be informative. The reduced drug liking in people with childhood adversities appears to be at odds with their overall greater risk of addiction (Dube et al., 2003). It is possible that stimulant drugs provide other benefits to people with adversities that motivate continued use. Leading motivations reported by young people who use stimulants were to improve functioning (to stay awake, and to get more energy), while the motivations for opioid use were to feel good and get high, to relieve pain and to relax (Drazdowski et al., 2020). For people with childhood adversities who typically report mental, physical and social challenges (Carlyle et al., 2021), the use of stimulants may be more driven by these functional (rather than pleasurable) effects. A recent experimental study showed that methamphetamine acutely increased feelings of connectedness after a conversation with a stranger in healthy volunteers (Molla et al., 2023). People with childhood adversities reported more social challenges, including loneliness (Carlyle et al., 2021, 2023) and less social support (Carlyle et al., 2021), where the subjective effects of stimulant drugs may be deemed as more positive when used in a social context.

Another perspective for the reduced subjective reward towards stimulants in this study is that participants with more childhood adversities yet no psychiatric or substance use problems (as pre-selected during screening) could reflect a particularly resilient population that does not represent all individuals exposed to childhood adversity. However, individuals with greater childhood adversities (particularly with high emotional neglect – a prevalent trauma subtype in this study) without a clinical diagnosis are more likely to show minimisation or denial when reporting emotionally charged events (MacDonald et al., 2016), which could have contributed to more conservative reporting of subjective stimulant effects. We note that such a pattern was not observed by Carlyle et al. (2021). The current study assessed the cumulative impact of childhood adversities across subscales of abuse and neglect due to the limited number of participants expected to report childhood adversities. However, deprivation-related (emotional and physical neglect) and threat-related adversities (emotional, physical and sexual abuses) have been linked to neural and behavioural differences in reward processing (Dennison et al., 2019; Liuzzi et al., 2023), where these group differences should be explored in a larger study with a greater number of participants with a broader range of adversities. Nonetheless, holistically understanding the function and contexts where drug effects are most rewarding for people with childhood adversities, as well as potential resilience factors, will be informative for targeted treatments.

Dampened responses to stimulants have previously been reported after clinical symptoms such as hypomanic-like experiences (Schepers et al., 2019), attention-deficit and hyperactivity disorder (ADHD; Marx, 1999) and anhedonia (Fan et al., 2022) – all of which are associated with childhood adversity. Stimulant medications are also believed to have calming effects for children and adults with ADHD (Harris et al., 2022). Epidemiological studies indicate a cumulative association between the number of adverse childhood events and increased odds of ADHD (Björkenstam et al., 2018), and it is possible that participants in this study with greater childhood adversities would have demonstrated more ADHD-like characteristics. However, an experimental study on the acute effects of ADHD medications in people with ADHD showed limited support for differences in feeling the stimulant effects between patients and controls (Kollins et al., 2009). Future studies should address how mood, hedonic capacity and ADHD-related symptoms separately as well as together with childhood adversities may influence responses to psychoactive drugs.

There are several strengths and limitations to this study. The included participant samples showed variable, yet comparatively low levels of adversity (only 10% had high adversity scores). A similar distribution of childhood adversity scores was observed in the previous studies with amphetamine administration (Oswald et al., 2014) and surgical opioid administration (Carlyle et al., 2023), but not in the morphine study (Carlyle et al., 2021). The relative lack of severe childhood adversity limits the generalisability of the findings. The current studies were among healthy participants with no psychiatric difficulties, which likely underestimates the true effect of childhood adversity – particularly for those with psychiatric morbidities. This also may limit the generalisability of the findings to more complex groups, i.e, with addictions and/or post-traumatic stress disorders, where longitudinal datasets would provide insights on whether and how early subjective drug effects are linked to continued use. All included studies measured the severity of childhood adversity using the CTQ, which is highly validated and eased between-study comparisons. However, the CTQ lacks information on potentially relevant characteristics such as the chronicity and age(s) of adversity, which should be explored further. In addition, there was no available data on subsequent or recent adverse events that could additionally impact subjective drug effects. Effect sizes were also expected to be small and a likely lack of statistical power was a significant limitation, though less so in the pooled analysis of the stimulant studies, where each participant completed four sessions. Grouping by sex further limited the sample sizes and impeded the ability to additionally explore divergent effects of sex that are frequently reported in the animal literature. While the primary analyses did not support sex-related differences in subjective effects, both exploratory sensitivity analyses and the bootstrapped confidence intervals for stimulant effects indicated that this non-significant sex effect may be less robust (SM6 and SM7).

## Conclusion

We find that even low levels of childhood adversities may have enduring impacts on the subjective experience of stimulant drugs. Possible avenues for further research include understanding how the context and function of drug use may differ for people with childhood adversities, in attempt to mechanistically probe the link between childhood adversity and addiction risk known from epidemiology. A deeper understanding on the initial motivation for substance use, and the specific conditions under which substance use is maintained, could inform novel preventions and early interventions to reduce adversity-related risks.

## Supplemental Material

sj-docx-1-jop-10.1177_02698811241268892 – Supplemental material for Impact of childhood adversity on acute subjective effects of stimulant and opioid drugs: Evidence from placebo-controlled studies in healthy volunteers Supplemental material, sj-docx-1-jop-10.1177_02698811241268892 for Impact of childhood adversity on acute subjective effects of stimulant and opioid drugs: Evidence from placebo-controlled studies in healthy volunteers by Molly Carlyle, Harriet de Wit and Siri Leknes in Journal of Psychopharmacology
