# Supplementary material for: Impact of childhood adversity on acute subjective effects of stimulant and opioid drugs: Evidence from placebo-controlled studies in healthy volunteers
Source: J Psychopharmacol. 2024 Aug 8;38(11):986–97. doi: 10.1177/02698811241268892 (PMC11528953; doi:10.1177/02698811241268892)
Supplement: sj-docx-1-jop-10.1177_02698811241268892 – Supplemental material for Impact of childhood adversity on acute subjective effects of stimulant and opioid drugs: Evidence from placebo-controlled studies in healthy volunteers [file sj-docx-1-jop-10.1177_02698811241268892.docx]

**Supplementary Materials**

Impact of childhood adversity on acute subjective effects of stimulant and opioid drugs: Evidence from placebo-controlled studies in healthy volunteers

**Contents:**

[SM1: CTQ scale established cut-off 2](#_Toc166491415)

[SM2 Table 1: Tests comparing responses to methamphetamine and d-amhetamine before data pooling 3](#_Toc166491416)

[SM3 Table 1. Random intercept multilevel model outcomes for the effect of childhood adversity on the subjective stimulant effects (controlling for study, age, and sex). 4](#_Toc166491417)

[SM3 Table 2. The CTQ × Drug × Time interaction for each time measured for feel effect, like effects, and feel high. 5](#_Toc166491418)

[SM4 Table 1. Random intercept multilevel model outcomes for the effect of childhood adversity on the buprenorphine effects (controlling for age and sex). 6](#_Toc166491419)

[SM4 Table 2. The CTQ × Time interaction for each time measured for feel effect, dislike effects, and feel high from buprenorphine. 7](#_Toc166491420)

[SM5 Table 1. Sensitivity analyses excluding timepoints 15, 90, 150, and 180 that were not possible to match across the studies. 8](#_Toc166491421)

[SM5 Table 2. Sensitivity analyses using linearly imputed data for timepoints 15, 90, 150, and 180 minutes that were not possible to match across the studies. 9](#_Toc166491422)

[SM11. Main findings for stimulants divided by gender 10](#_Toc166491423)

[Table SM7: Bootstrapped estimates and confidence intervals for stimulant models. 12](#_Toc166491424)

[Table SM8: Bootstrapped estimates and confidence intervals for buprenorphine models 13](#_Toc166491425)

[Table SM9. RI-MLM on the effects of CTQ score on heart rate (bpm) after drug administration. 14](#_Toc166491426)

[Figure SM10. Group differences in drug responses (average placebo-adjusted AUC) 15](file:///C:\Users\mollyca\UiO%20Dropbox\Molly%20Carlyle\DeWit%20Secondary%20data\Manuscript%20document\Journal%20of%20Psychopharmacology\Revise%20&%20resubmit\SupplementaryMaterials_v2_20240415.docx#_Toc166491427)

[16](#_Toc166491428)

[Figure SM11. Drug responses (AUC) divided by sex and childhood adversity group across all studies 16](file:///C:\Users\mollyca\UiO%20Dropbox\Molly%20Carlyle\DeWit%20Secondary%20data\Manuscript%20document\Journal%20of%20Psychopharmacology\Revise%20&%20resubmit\SupplementaryMaterials_v2_20240415.docx#_Toc166491429)

# SM1: CTQ scale established cut-off

Predetermined cut-offs to categorise severity scores:

**Emotional Abuse: Items 3,8,14,18,25**

None = 5-8; Low = 9-12; Moderate = 13-15; Severe = 16+

**Physical Abuse: Items 9,11,12,15,17**

None = 5-7; Low = 8-9; Moderate = 10-12; Severe = 13+

**Sexual Abuse: Items 20,21,23,24,27**

None = 5; Low = 6-7; Moderate = 8-12; Severe = 13+

**Emotional Neglect: Items 5,7,13,19, 28**

None = 5-9; Low = 10-14; Moderate = 15-17; Severe = 18+

**Physical Neglect: Items 1,2,4,6,26**

None = 5-7; Low = 8-9; Moderate = 10-12; Severe = 13+

**Citations:**

Bernstein, D. P., & Fink, L. (1998). *Childhood Trauma Questionnaire. A retrospective self-report. Manual*. San Antonio. TX: The Psychological Corporation. Harcourt Brace & Company

Bernstein, D. P., & Fink, L. (2011). *Childhood Trauma Questionnaire. A retrospective self-report. Manual – Svensk version [Swedish version]*. Stockholm: Pearson Assessment

Hagborg, J.M., Kalin, T. & Gerdner, A. The Childhood Trauma Questionnaire—Short Form (CTQ-SF) used with adolescents – methodological report from clinical and community samples. *Journ Child Adol Trauma* **15**, 1199–1213 (2022). <https://doi.org/10.1007/s40653-022-00443-8>

# SM2 Table 1: Tests comparing responses to methamphetamine and d-amhetamine before data pooling

|  | **MD** | **Mean meth.** | **Mean**  **d-Amph** | **Test statistic** | **p** | **CI low** | **CI high** | **Method** | **Alternative** |
| --- | --- | --- | --- | --- | --- | --- | --- | --- | --- |
| Feel | 6.06 | 15.07 | 21.14 | -1.59 | 0.12 | -13.68 | 1.55 | Welch Two Sample t-test | Two.sided |
| Like | 2.68 | 29.02 | 31.7 | -0.48 | 0.63 | -13.81 | 8.45 |  |  |
| High | 3.91 | 14.59 | 18.49 | -1.06 | 0.29 | -11.3 | 3.49 |  |  |
| Dislike | 0.01 | -0.74 | -0.75 | 0 | >.99 | -7.71 | 7.73 |  |  |
| More | 3.96 | 33.38 | 29.42 | 0.64 | 0.52 | -8.32 | 16.24 |  |  |

*Notes*. Analyses of differences in overall stimulant responses to methamphetamine and d-amphetamine prior to pooling for the main analyses. These preliminary analyses were between subject t-tests on overall placebo-controlled area-under-the-curve on the drug responses. These non-significant differences align with the existing literature that states comparable subjective and behavioural effects e.g., citation: Martin WR, Sloan JW, Sapira JD, Jasinski DR. Physiologic, subjective, and behavioral effects of amphetamine, methamphetamine, ephedrine, phenmetrazine, and methylphenidate in man. Clinical Pharmacology & Therapeutics. 1971;12(2part1):245–58.

| **SM3 Table 1.** Random intercept multilevel model outcomes for the effect of childhood adversity on the subjective stimulant effects (controlling for study, age, and sex). | | | | | | | | | | | | | | | | |  |
| --- | --- | --- | --- | --- | --- | --- | --- | --- | --- | --- | --- | --- | --- | --- | --- | --- | --- |
|  | **Feel effects** | | | **Like effects** | | | **Dislike effects** | | | **Feel high** | | | **Want more** | | | |  |
| *Predictors* | *Estimate* | *95% CIs* | *p* | *Estimate* | *95% CIs* | *p* | *Estimate* | *95% CIs* | *p* | *Estimate* | *95% CIs* | *p* | *Estimate* | *95% CIs* | *p* | |  |
| Time | -0.20 | -1.55 – 1.16 | .776 | -0.05 | -1.74 – 1.63 | .950 | 0.97 | -0.39 – 2.33 | .161 | -0.06 | -1.28 – 1.16 | .927 | 1.10 | -0.49 – 2.69 | .176 | |  |
| CTQ score | -0.07 | -0.36 – 0.22 | .635 | 0.00 | -0.36 – 0.36 | .988 | -0.08 | -0.38 – 0.22 | .606 | -0.10 | -0.37 – 0.18 | .503 | -0.04 | -0.40 – 0.32 | .838 | |  |
| Drug | 1.56 | -8.70 – 11.83 | .765 | 4.19 | -8.59 – 16.96 | .520 | -4.41 | -14.69 – 5.86 | .400 | -1.33 | -10.59 – 7.92 | .777 | -2.57 | -14.64 – 9.50 | .676 | |  |
| Study | 1.59 | -3.58 – 6.76 | .547 | -0.58 | -7.04 – 5.87 | .859 | 0.01 | -5.46 – 5.48 | .997 | 1.54 | -3.63 – 6.70 | .560 | -2.28 | -9.02 – 4.46 | .507 | |  |
| Age | 0.25 | -0.43 – 0.93 | .471 | -0.28 | -1.13 – 0.57 | .512 | 0.37 | -0.35 – 1.09 | .319 | 0.46 | -0.22 – 1.15 | .181 | -0.21 | -1.10 – 0.67 | .637 | |  |
| Sex | 2.91 | -2.09 – 7.90 | .254 | -0.75 | -6.99 – 5.49 | .814 | 3.70 | -1.58 – 8.99 | .169 | 1.85 | -3.15 – 6.85 | .468 | -3.40 | -9.92 – 3.12 | .307 | |  |
| CTQ*Time | 0.01 | -0.03 – 0.05 | .648 | 0.02 | -0.03 – 0.06 | .503 | -0.00 | -0.04 – 0.03 | .858 | 0.01 | -0.03 – 0.04 | .729 | -0.01 | -0.05 – 0.03 | .636 | |  |
| Drug*Time | 5.34 | 3.45 – 7.24 | **<.001** | 6.88 | 4.52 – 9.23 | **<.001** | 1.46 | -0.44 – 3.35 | .132 | 4.41 | 2.70 – 6.12 | **<.001** | 6.40 | 4.17 – 8.63 | **<.001** | |  |
| CTQ*Drug | 0.02 | -0.26 – 0.30 | .886 | 0.03 | -0.32 – 0.38 | .866 | 0.04 | -0.24 – 0.32 | .789 | 0.13 | -0.12 – 0.38 | .309 | 0.13 | -0.20 – 0.45 | .453 | |  |
| CTQ*Drug*Time | -0.07 | -0.12 – -0.02 | **.009** | -0.07 | -0.13 – -0.00 | **.038** | -0.03 | -0.09 – 0.02 | .220 | -0.06 | -0.10 – -0.01 | **.020** | -0.04 | -0.10 – 0.03 | .249 | |  |
| **Random Effects** | | | | | | | | | | | | | | | | | |
| σ^2^ | 354.57 | | | 549.13 | | | 355.12 | | | 288.11 | | | 490.20 | | | |  |
| τ_00_ | 33.68 _Session:ID_ | | | 26.36 _Session:ID_ | | | 21.65 _Session:ID_ | | | 19.57 _Session:ID_ | | | 29.40 _Session:ID_ | | | |  |
|  | 96.06 _ID_ | | | 162.86 _ID_ | | | 116.88 _ID_ | | | 105.41 _ID_ | | | 181.46 _ID_ | | | |  |
| ICC | 0.27 | | | 0.26 | | | 0.28 | | | 0.30 | | | 0.30 | | | |  |
| N | 2 _Session_ | | | 2 _Session_ | | | 2 _Session_ | | | 2 _Session_ | | | 2 _Session_ | | | |  |
|  | 84 _ID_ | | | 84 _ID_ | | | 84 _ID_ | | | 84 _ID_ | | | 84 _ID_ | | | |  |
| Obs. | 2420 | | | 2420 | | | 2420 | | | 2420 | | | 2420 | | | |  |
| Marginal R^2^ /Conditional R^2^ | 0.196 / 0.412 | | | 0.278 / 0.463 | | | 0.030 / 0.302 | | | 0.185 / 0.431 | | | 0.308 / 0.516 | | | |  |
| *Notes*. ‘Drug’ is a factor variable with placebo coded as ‘1’ and stimulant coded as ‘2’. ‘Time’ is a numerical variable reflecting 0 (baseline), and 15, 30, 65, 90, 117, 150, 180, 205 minutes post-drug administration. Obs – observations, CTQ – childhood trauma questionnaire. Drug – stimulant or placebo. Study –methamphetamine or d-amphetamine. | | | | | | | | | | | | | | | |  |  |

| **SM3 Table 2**. The CTQ × Drug × Time interaction for each time measured for feel effect, like effects, and feel high. | | | | | | | | | |
| --- | --- | --- | --- | --- | --- | --- | --- | --- | --- |
|  | **Feel effects** | | | **Like effects** | | | **Feel high** | | |
|  | *Estimates* | *CI* | *p* | *Estimates* | *CI* | *p* | *Estimates* | *CI* | *p* |
| 15 minutes | -0.10 | -0.61 – 0.40 | 0.689 | -0.31 | -0.95 – 0.33 | 0.342 | 0.00 | -0.46 – 0.47 | 0.990 |
| 30 minutes | 0.05 | -0.40 – 0.49 | 0.837 | 0.29 | -0.28 – 0.86 | 0.317 | 0.27 | -0.14 – 0.69 | 0.200 |
| 65 minutes | -0.06 | -0.50 – 0.39 | 0.804 | 0.09 | -0.48 – 0.66 | 0.762 | 0.10 | -0.32 – 0.51 | 0.649 |
| 90 minutes | -1.09 | -1.77 – -0.41 | **0.002** | -0.77 | -1.64 – 0.09 | 0.080 | -0.82 | -1.45 – -0.19 | **0.011** |
| 117 minutes | -0.57 | -1.02 – -0.12 | **0.012** | -0.36 | -0.93 – 0.21 | 0.215 | -0.26 | -0.67 – 0.16 | 0.222 |
| 150 minutes | -1.23 | -1.91 – -0.55 | **<0.001** | -0.95 | -1.82 – -0.09 | **0.030** | -0.89 | -1.52 – -0.26 | **0.005** |
| 180 minutes | -1.07 | -1.75 – -0.39 | **0.002** | -1.16 | -2.02 – -0.30 | **0.008** | -0.80 | -1.43 – -0.17 | **0.012** |
| 205 minutes | -0.18 | -0.63 – 0.27 | 0.429 | -0.26 | -0.83 – 0.31 | 0.363 | -0.11 | -0.53 – 0.31 | 0.604 |
| *Notes.* To identify the specific timepoints driving the significant interaction, ‘time’ was inserted as a factor and re-ran in the MLMs. As such, baseline has been selected as the contrast time, and therefore the estimates and p-values reported here must be interpreted in relation to baseline. Significance p<.05 is indicated by bolded text. | | | | | | | | | |

| **SM4 Table 1.** Random intercept multilevel model outcomes for the effect of childhood adversity on the buprenorphine effects (controlling for age and sex). | | | | | | | | | | | | | | | |
| --- | --- | --- | --- | --- | --- | --- | --- | --- | --- | --- | --- | --- | --- | --- | --- |
|  | Feel effects | | | Like effects | | | Dislike effects | | | Feel high | | | Want more | | |
| *Predictors* | *Estimates* | *95% CIs* | *p* | *Estimates* | *95% CIs* | *p* | *Estimates* | *95% CIs* | *p* | *Estimates* | *95% CIs* | *p* | *Estimates* | *95% CIs* | *p* |
| Time | 12.12 | 4.83 – 19.40 | **0.001** | 1.47 | -8.01 – 10.96 | 0.760 | 13.50 | 4.43 – 22.58 | **0.004** | 4.89 | 0.12 – 9.66 | **0.044** | 6.36 | -1.68 – 14.41 | 0.121 |
| CTQ score | 0.37 | -0.31 – 1.06 | 0.286 | -0.28 | -1.18 – 0.63 | 0.549 | 0.69 | -0.18 – 1.56 | 0.121 | 0.04 | -0.44 – 0.53 | 0.855 | 0.11 | -0.71 – 0.93 | 0.788 |
| Drug | 5.92 | -28.26 – 40.09 | 0.734 | -14.43 | -58.92 – 30.07 | 0.524 | 0.35 | -42.21 – 42.91 | 0.987 | 9.18 | -13.17 – 31.54 | 0.420 | 8.22 | -29.54 – 45.97 | 0.669 |
| Age | -0.30 | -1.05 – 0.45 | 0.436 | -0.24 | -1.25 – 0.77 | 0.640 | -0.53 | -1.54 – 0.48 | 0.306 | 0.05 | -0.59 – 0.68 | 0.887 | -0.20 | -1.27 – 0.88 | 0.719 |
| Sex | 4.17 | -3.41 – 11.74 | 0.280 | -1.56 | -11.78 – 8.66 | 0.764 | 0.81 | -9.38 – 11.00 | 0.876 | -0.32 | -6.75 – 6.10 | 0.921 | 4.01 | -6.82 – 14.83 | 0.467 |
| CTQ*Time | -0.19 | -0.38 – -0.00 | **0.045** | -0.01 | -0.25 – 0.24 | 0.954 | -0.15 | -0.38 – 0.09 | 0.227 | -0.06 | -0.19 – 0.06 | 0.310 | -0.12 | -0.33 – 0.09 | 0.246 |
| Drug*Time | -9.67 | -19.97 – 0.64 | 0.066 | 3.81 | -9.60 – 17.23 | 0.577 | -11.44 | -24.27 – 1.40 | 0.080 | -4.93 | -11.67 – 1.81 | 0.151 | -2.90 | -14.28 – 8.49 | 0.617 |
| CTQ*Drug | 0.01 | -0.88 – 0.90 | 0.981 | 0.34 | -0.82 – 1.50 | 0.565 | 0.26 | -0.85 – 1.37 | 0.644 | -0.14 | -0.72 – 0.44 | 0.631 | -0.32 | -1.30 – 0.66 | 0.524 |
| CTQ*Drug*Time | 0.11 | -0.15 – 0.38 | 0.400 | -0.08 | -0.43 – 0.26 | 0.633 | 0.16 | -0.18 – 0.49 | 0.358 | 0.05 | -0.12 – 0.23 | 0.564 | 0.11 | -0.19 – 0.40 | 0.485 |
| Random Effects | | | | | | | | | | | | | | | |
| σ^2^ | 355.65 | | | 602.76 | | | 551.55 | | | 152.16 | | | 434.01 | | |
| τ_00_ | 66.38 _SubjectNum_ | | | 125.48 _SubjectNum_ | | | 129.49 _SubjectNum_ | | | 58.24 _SubjectNum_ | | | 164.89 _SubjectNum_ | | |
| ICC | 0.16 | | | 0.17 | | | 0.19 | | | 0.28 | | | 0.28 | | |
| N | 34 _SubjectNum_ | | | 34 _SubjectNum_ | | | 34 _SubjectNum_ | | | 34 _SubjectNum_ | | | 34 _SubjectNum_ | | |
| Observations | 340 | | | 340 | | | 340 | | | 340 | | | 340 | | |
| Marginal R^2^ / Conditional R^2^ | 0.130 / 0.267 | | | 0.023 / 0.191 | | | 0.174 / 0.331 | | | 0.070 / 0.328 | | | 0.042 / 0.306 | | |

| **SM4 Table 2.** The CTQ × Time interaction for each time measured for feel effect, dislike effects, and feel high from buprenorphine. | | | | | | | | | |
| --- | --- | --- | --- | --- | --- | --- | --- | --- | --- |
|  | **Feel effects** | | | **Dislike effects** | | | **Feel high** | | |
| *Predictors* | *Estimates* | *95% CIs* | *p* | *Estimates* | *95% CIs* | *p* | *Estimates* | *95% CIs* | *p* |
| 30 minutes | 22.45 | -6.43 – 51.33 | 0.127 | 27.58 | -9.78 – 64.94 | 0.147 | 20.04 | 0.41 – 39.68 | **0.045** |
| 60 minutes | 33.79 | 4.91 – 62.67 | **0.022** | 6.51 | -30.85 – 43.86 | 0.732 | 13.28 | -6.35 – 32.92 | 0.184 |
| 180 minutes | 50.21 | 21.33 – 79.09 | **0.001** | 37.05 | -0.31 – 74.41 | 0.052 | 24.44 | 4.81 – 44.08 | **0.015** |
| 210 minutes | 46.71 | 17.83 – 75.59 | **0.002** | 62.77 | 25.42 – 100.13 | **0.001** | 22.25 | 2.61 – 41.88 | **0.027** |
| CTQ*30 minutes | 0.04 | -0.71 – 0.79 | 0.924 | -0.28 | -1.25 – 0.69 | 0.574 | -0.21 | -0.72 – 0.30 | 0.427 |
| CTQ*60 minutes | -0.18 | -0.93 – 0.57 | 0.635 | 0.45 | -0.52 – 1.42 | 0.360 | 0.03 | -0.48 – 0.54 | 0.899 |
| CTQ*180 minutes | -0.59 | -1.34 – 0.16 | 0.123 | -0.01 | -0.98 – 0.96 | 0.988 | -0.26 | -0.77 – 0.25 | 0.315 |
| CTQ*210 minutes | -0.65 | -1.41 – 0.10 | 0.088 | -0.86 | -1.83 – 0.11 | 0.082 | -0.29 | -0.80 – 0.22 | 0.260 |

*Notes.* To identify the specific timepoints driving the significant interaction, ‘Time’ was inserted as a factor variable, where baseline has been selected as the contrast time and estimates and p-values reported here must be interpreted in relation to baseline. Significance p<.05 is indicated by bolded text.

| **SM5 Table 1.** Sensitivity analyses excluding timepoints 15, 90, 150, and 180 that were not possible to match across the studies. | | | | | | | | | | | | | | | |  |
| --- | --- | --- | --- | --- | --- | --- | --- | --- | --- | --- | --- | --- | --- | --- | --- | --- |
|  | **Feel effects** | | | **Like effects** | | | **Dislike effects** | | | **Feel high** | | | **Want more** | | | |
| *Predictors* | *E* | *95% CIs* | *p* | *E* | *95% CIs* | *p* | *E* | *95% CIs* | *p* | *E* | *95% CIs* | *p* | *E* | *95% CIs* | *p* | |
| Time | 0.54 | -1.02 – 2.10 | 0.496 | 0.34 | -1.60 – 2.29 | 0.729 | 1.51 | -0.05 – 3.07 | 0.058 | 0.43 | -0.96 – 1.81 | 0.547 | 1.39 | -0.42 – 3.19 | 0.132 | |
| CTQ score | -0.03 | -0.33 – 0.26 | 0.818 | -0.06 | -0.43 – 0.31 | 0.763 | -0.08 | -0.39 – 0.24 | 0.634 | -0.07 | -0.35 – 0.21 | 0.613 | -0.04 | -0.41 – 0.33 | 0.837 | |
| Drug | 1.11 | -10.65 – 12.87 | 0.853 | 0.58 | -14.12 – 15.27 | 0.939 | -3.62 | -15.38 – 8.15 | 0.547 | -2.56 | -13.03 – 7.92 | 0.632 | -4.00 | -17.62 – 9.61 | 0.564 | |
| Study | -0.47 | -5.17 – 4.23 | 0.845 | -2.45 | -8.47 – 3.57 | 0.425 | -0.10 | -5.37 – 5.18 | 0.971 | -0.43 | -5.10 – 4.24 | 0.857 | -3.68 | -10.09 – 2.73 | 0.260 | |
| Age | 0.30 | -0.32 – 0.92 | 0.339 | -0.17 | -0.96 – 0.63 | 0.679 | 0.42 | -0.28 – 1.11 | 0.242 | 0.49 | -0.12 – 1.11 | 0.117 | -0.13 | -0.97 – 0.72 | 0.766 | |
| Sex | 2.47 | -2.10 – 7.05 | 0.289 | -0.89 | -6.75 – 4.96 | 0.765 | 3.69 | -1.44 – 8.82 | 0.159 | 1.67 | -2.87 – 6.21 | 0.470 | -2.86 | -9.09 – 3.37 | 0.368 | |
| CTQ*Time | -0.01 | -0.05 – 0.04 | 0.765 | 0.01 | -0.04 – 0.07 | 0.637 | -0.01 | -0.05 – 0.03 | 0.589 | -0.01 | -0.04 – 0.03 | 0.769 | -0.02 | -0.07 – 0.03 | 0.529 | |
| Drug*Time | 3.99 | 1.79 – 6.20 | **<.001** | 6.02 | 3.27 – 8.77 | **<.001** | 1.04 | -1.17 – 3.24 | 0.356 | 3.44 | 1.48 – 5.40 | **.001** | 6.01 | 3.46 – 8.56 | **<.001** | |
| CTQ*Drug | 0.06 | -0.26 – 0.38 | 0.714 | 0.17 | -0.24 – 0.57 | 0.417 | 0.04 | -0.29 – 0.36 | 0.826 | 0.17 | -0.11 – 0.46 | 0.238 | 0.19 | -0.18 – 0.57 | 0.307 | |
| CTQ*Drug*Time | -0.04 | -0.10 – 0.02 | 0.158 | -0.06 | -0.13 – 0.02 | 0.134 | -0.02 | -0.08 – 0.04 | 0.432 | -0.04 | -0.09 – 0.02 | 0.197 | -0.03 | -0.10 – 0.04 | 0.360 | |
| Random Effects | | | | | | | | | | | | | | | |  |
| σ^2^ | 372.36 | | | 581.43 | | | 372.55 | | | 295.43 | | | 499.10 | | | |
| τ_00_ | 15.80 _Session:ID_ | | | 10.01 _Session:ID_ | | | 7.91 _Session:ID_ | | | 10.22 _Session:ID_ | | | 11.65 _Session:ID_ | | | |
|  | 78.30 _ID_ | | | 137.76 _ID_ | | | 109.34 _ID_ | | | 83.36 _ID_ | | | 163.93 _ID_ | | | |
| ICC | 0.20 | | | 0.20 | | | 0.24 | | | 0.24 | | | 0.26 | | | |
| N | 2 _Session_ | | | 2 _Session_ | | | 2 _Session_ | | | 2 _Session_ | | | 2 _Session_ | | | |
|  | 84 _ID_ | | | 84 _ID_ | | | 84 _ID_ | | | 84 _ID_ | | | 84 _ID_ | | | |
| Observations | 1670 | | | 1670 | | | 1670 | | | 1670 | | | 1670 | | | |
| Marginal R^2^ / Conditional R^2^ | 0.159 / 0.329 | | | 0.251 / 0.403 | | | 0.038 / 0.268 | | | 0.158 / 0.361 | | | 0.299 / 0.481 | | | |

| **SM5 Table 2.** Sensitivity analyses using linearly imputed data for timepoints 15, 90, 150, and 180 minutes that were not possible to match across the studies. | | | | | | | | | | | | | | | |
| --- | --- | --- | --- | --- | --- | --- | --- | --- | --- | --- | --- | --- | --- | --- | --- |
|  | **Feel effects** | | | **Like effects** | | | **Dislike effects** | | | **Feel high** | | | **Want more** | | |
| *Predictors* | *E* | *95% CIs* | *p* | *E* | *95% CIs* | *p* | *E* | *95% CIs* | *p* | *E* | *95% CIs* | *p* | *E* | *95% CIs* | *p* |
| Time | 0.27 | -0.87 – 1.41 | 0.642 | 0.22 | -1.20 – 1.64 | 0.762 | 1.11 | -0.03 – 2.26 | 0.057 | 0.41 | -0.63 – 1.44 | 0.441 | 1.18 | -0.17 – 2.52 | 0.087 |
| CTQ score | -0.02 | -0.30 – 0.25 | 0.865 | 0.02 | -0.33 – 0.36 | 0.920 | -0.07 | -0.36 – 0.21 | 0.617 | -0.06 | -0.32 – 0.21 | 0.673 | -0.02 | -0.38 – 0.33 | 0.897 |
| Drug | 0.58 | -8.51 – 9.67 | 0.901 | 1.16 | -10.13 – 12.45 | 0.840 | -3.65 | -12.76 – 5.47 | 0.433 | -2.05 | -10.26 – 6.17 | 0.625 | -4.88 | -15.60 – 5.84 | 0.372 |
| Study | 1.05 | -3.96 – 6.06 | 0.681 | -1.37 | -7.76 – 5.03 | 0.675 | -0.70 | -6.13 – 4.73 | 0.800 | 0.89 | -4.08 – 5.87 | 0.725 | -2.75 | -9.51 – 4.01 | 0.426 |
| Age | 0.34 | -0.32 – 1.00 | 0.318 | -0.25 | -1.10 – 0.59 | 0.555 | 0.38 | -0.34 – 1.09 | 0.302 | 0.53 | -0.12 – 1.19 | 0.112 | -0.20 | -1.09 – 0.70 | 0.667 |
| Sex | 2.75 | -2.13 – 7.63 | 0.269 | -0.77 | -6.98 – 5.44 | 0.808 | 3.82 | -1.46 – 9.10 | 0.156 | 1.57 | -3.27 – 6.41 | 0.525 | -3.20 | -9.77 – 3.37 | 0.340 |
| CTQ*Time | -0.00 | -0.03 – 0.03 | 0.848 | 0.01 | -0.03 – 0.05 | 0.716 | -0.01 | -0.04 – 0.02 | 0.690 | -0.01 | -0.03 – 0.02 | 0.677 | -0.02 | -0.05 – 0.02 | 0.389 |
| Drug*Time | 4.73 | 3.11 – 6.34 | **<.001** | 6.64 | 4.63 – 8.65 | **<.001** | 1.38 | -0.24 – 3.00 | 0.094 | 3.83 | 2.37 – 5.29 | **<.001** | 6.43 | 4.53 – 8.33 | **<.001** |
| CTQ*Drug | 0.03 | -0.22 – 0.28 | 0.830 | 0.07 | -0.24 – 0.38 | 0.661 | 0.03 | -0.22 – 0.28 | 0.822 | 0.13 | -0.10 – 0.35 | 0.273 | 0.16 | -0.14 – 0.45 | 0.296 |
| CTQ*Drug*Time | -0.05 | -0.09 – -0.01 | **0.025** | -0.05 | -0.11 – 0.00 | **0.050** | -0.03 | -0.08 – 0.01 | 0.168 | -0.04 | -0.08 – 0.00 | **0.070** | -0.03 | -0.08 – 0.02 | 0.275 |
| Random Effects | | | | | | | | | | | | | | | |
| σ^2^ | 324.48 | | | 500.74 | | | 326.33 | | | 264.99 | | | 451.22 | | |
| τ_00_ | 41.52 _Session:ID_ | | | 49.13 _Session:ID_ | | | 36.25 _Session:ID_ | | | 26.76 _Session:ID_ | | | 42.60 _Session:ID_ | | |
|  | 89.60 _ID_ | | | 155.40 _ID_ | | | 112.66 _ID_ | | | 96.81 _ID_ | | | 183.12 _ID_ | | |
| ICC | 0.29 | | | 0.29 | | | 0.31 | | | 0.32 | | | 0.33 | | |
| N | 2 _Session_ | | | 2 _Session_ | | | 2 _Session_ | | | 2 _Session_ | | | 2 _Session_ | | |
|  | 84 _ID_ | | | 84 _ID_ | | | 84 _ID_ | | | 84 _ID_ | | | 84 _ID_ | | |
| Observations | 3024 | | | 3024 | | | 3024 | | | 3024 | | | 3024 | | |
| Marginal R^2^ / Conditional R^2^ | 0.192 / 0.425 | | | 0.284 / 0.492 | | | 0.032 / 0.335 | | | 0.185 / 0.444 | | | 0.321 / 0.547 | | |

## SM11. Main findings for stimulants divided by gender

**Table 1:** feel effects (only fixed effects reported)

|  | Feel - female | | | Feel - male | | |
| --- | --- | --- | --- | --- | --- | --- |
| *Predictors* | *Estimates* | *95% CIs* | *p* | *Estimates* | *95% CIs* | *p* |
| Time | -0.47 | -2.25 – 1.32 | 0.607 | 0.46 | -1.71 – 2.63 | 0.678 |
| CTQ | -0.06 | -0.45 – 0.33 | 0.772 | -0.07 | -0.51 – 0.37 | 0.746 |
| Drug | -1.18 | -14.76 – 12.40 | 0.865 | 5.46 | -10.88 – 21.81 | 0.512 |
| Study | 2.78 | -5.58 – 11.14 | 0.514 | 0.48 | -5.51 – 6.47 | 0.875 |
| Age | 0.10 | -1.24 – 1.45 | 0.878 | 0.29 | -0.38 – 0.96 | 0.394 |
| Time*CTQ | 0.02 | -0.03 – 0.07 | 0.432 | -0.01 | -0.08 – 0.05 | 0.650 |
| Time*Drug | 6.25 | 3.76 – 8.75 | **<0.001** | 3.90 | 0.86 – 6.94 | **0.012** |
| CTQ*Drug | 0.07 | -0.28 – 0.43 | 0.682 | -0.07 | -0.53 – 0.40 | 0.780 |
| Time*CTQ*Drug | -0.09 | -0.16 – -0.02 | **0.008** | -0.03 | -0.12 – 0.05 | 0.456 |
| Observations | 1333 | | | 1087 | | |

**Table 2:** Like effects (only fixed effects reported)

|  | like - female | | | Like - male | | |
| --- | --- | --- | --- | --- | --- | --- |
| *Predictors* | *Estimates* | *95% CIs* | *p* | *Estimates* | *95% CIs* | *p* |
| Time | -0.60 | -2.76 – 1.56 | 0.586 | 1.02 | -1.80 – 3.84 | 0.477 |
| CTQ | 0.11 | -0.35 – 0.57 | 0.625 | -0.25 | -0.84 – 0.34 | 0.412 |
| Drug | 5.22 | -11.21 – 21.65 | 0.533 | 1.92 | -19.28 – 23.12 | 0.859 |
| Study | -0.67 | -10.37 – 9.02 | 0.891 | -0.76 | -9.16 – 7.64 | 0.860 |
| Age | -0.36 | -1.92 – 1.20 | 0.652 | -0.23 | -1.17 – 0.71 | 0.628 |
| Time*CTQ | 0.03 | -0.03 – 0.09 | 0.287 | -0.02 | -0.10 – 0.07 | 0.694 |
| Time*Drug | 7.03 | 4.01 – 10.05 | **<0.001** | 6.48 | 2.54 – 10.41 | **0.001** |
| CTQ*Drug | -0.00 | -0.43 – 0.42 | 0.990 | 0.10 | -0.50 – 0.71 | 0.742 |
| Time*CTQ*Drug | -0.07 | -0.15 – 0.01 | 0.069 | -0.05 | -0.17 – 0.06 | 0.345 |
| Observations | 1333 | | | 1087 | | |

**Table 3:** Feel high(only fixed effects reported)

|  | High - female | | | High - male | | |
| --- | --- | --- | --- | --- | --- | --- |
| *Predictors* | *Estimates* | *95% CIs* | *p* | *Estimates* | *95% CIs* | *p* |
| Time | -0.60 | -2.76 – 1.56 | 0.586 | 1.02 | -1.80 – 3.84 | 0.477 |
| CTQ | 0.11 | -0.35 – 0.57 | 0.625 | -0.25 | -0.84 – 0.34 | 0.412 |
| Drug | 5.22 | -11.21 – 21.65 | 0.533 | 1.92 | -19.28 – 23.12 | 0.859 |
| Study | -0.67 | -10.37 – 9.02 | 0.891 | -0.76 | -9.16 – 7.64 | 0.860 |
| Age | -0.36 | -1.92 – 1.20 | 0.652 | -0.23 | -1.17 – 0.71 | 0.628 |
| Time*CTQ | 0.03 | -0.03 – 0.09 | 0.287 | -0.02 | -0.10 – 0.07 | 0.694 |
| Time*Drug | 7.03 | 4.01 – 10.05 | **<0.001** | 6.48 | 2.54 – 10.41 | **0.001** |
| CTQ*Drug | -0.00 | -0.43 – 0.42 | 0.990 | 0.10 | -0.50 – 0.71 | 0.742 |
| Time*CTQ*Drug | -0.07 | -0.15 – 0.01 | 0.069 | -0.05 | -0.17 – 0.06 | 0.345 |
| Observations | 1333 | | | 1087 | | |

| **Table SM7:** Bootstrapped estimates and confidence intervals for stimulant models. | | | | | |  |
| --- | --- | --- | --- | --- | --- | --- |
|  | **Bootstrapped estimates** | | **Original model** | |  | |
|  | **B: Estimate** | **B: 95% CIs** | **Estimate** | **95% CIs** |  | |
| **Feel** |  |  |  |  |  | |
| *Time* | -0.14 | -1.55 – 1.13 | -0.2 | -1.55 – 1.16 |  | |
| *CTQ score* | -0.06 | -0.26 – 0.13 | -0.07 | -0.36 – 0.22 |  | |
| *Drug* | 1.67 | -8.55 – 11.38 | 1.56 | -8.70 – 11.83 |  | |
| *Study* | 1.59 | -0.19 – 3.39 | 1.59 | -3.58 – 6.76 |  | |
| *Age* | 0.25 | 0.03 – 0.47 | 0.25 | -0.43 – 0.93 |  | |
| *Sex* | 2.94 | 1.47 – 4.47 ^a^ | 2.91 | -2.09 – 7.90 |  | |
| *CTQ*Time* | 0.01 | -0.03 – 0.05 | 0.01 | -0.03 – 0.05 |  | |
| ***Drug*Time*** | **5.33** | **3.52 – 7.15** | **5.34** | **3.45 – 7.24** |  | |
| *CTQ*Drug* | 0.02 | -0.25 – 0.31 | 0.02 | -0.26 – 0.30 |  | |
| ***CTQ*Drug*Time*** | **-0.07** | **-0.12 – -0.02** | **-0.07** | **-0.12 – -0.02** |  | |
| **Like** |  |  |  |  |  | |
| *Time* | -0.03 | -1.76 – 1.59 | -0.05 | -1.74 – 1.63 |  | |
| *CTQ score* | 0 | -0.24 – 0.25 | 0 | -0.36 – 0.36 |  | |
| *Drug* | 3.86 | -8.53 – 17.47 | 4.19 | -8.59 – 16.96 |  | |
| *Study* | -0.66 | -2.85 – 1.45 | -0.58 | -7.04 – 5.87 |  | |
| *Age* | -0.26 | -0.56 – 0.03 | -0.28 | -1.13 – 0.57 |  | |
| *Sex* | -0.7 | -2.61 – 1.22 | -0.75 | -6.99 – 5.49 |  | |
| *CTQ*Time* | 0.02 | -0.03 – 0.06 | 0.02 | -0.03 – 0.06 |  | |
| ***Drug*Time*** | **6.93** | **4.44 – 9.41** | **6.88** | **4.52 – 9.23** |  | |
| *CTQ*Drug* | 0.04 | -0.35 – 0.39 | 0.03 | -0.32 – 0.38 |  | |
| ***CTQ*Drug*Time*** | **-0.07** | **-0.14 – 0** | **-0.07** | **-0.13 – -0.00** |  | |
| **Dislike** |  |  |  |  |  | |
| *Time* | 1 | -0.36 – 2.38 | 0.97 | -0.39 – 2.33 |  | |
| *CTQ score* | -0.08 | -0.27 – 0.13 | -0.08 | -0.38 – 0.22 |  | |
| *Drug* | -4.47 | -14.47 – 5.52 | -4.41 | -14.69 – 5.86 |  | |
| *Study* | **-0.1** | **-1.78 – 1.57** | 0.01 | -5.46 – 5.48 |  | |
| *Age* | **0.38** | **0.16 – 0.62 ^a^** | 0.37 | -0.35 – 1.09 |  | |
| *Sex* | **3.71** | **2.13 – 5.24 ^a^** | 3.7 | -1.58 – 8.99 |  | |
| *CTQ*Time* | 0 | -0.04 – 0.03 | 0 | -0.04 – 0.03 |  | |
| *Drug*Time* | 1.47 | -0.27 – 3.4 | 1.46 | -0.44 – 3.35 |  | |
| *CTQ*Drug* | 0.04 | -0.23 – 0.3 | 0.04 | -0.24 – 0.32 |  | |
| *CTQ*Drug*Time* | -0.03 | -0.09 – 0.02 | -0.03 | -0.09 – 0.02 |  | |
| **High** |  |  |  |  |  | |
| *Time* | -0.04 | -1.27 – 1.14 | -0.06 | -1.28 – 1.16 |  | |
| *CTQ score* | -0.09 | -0.28 – 0.08 | -0.1 | -0.37 – 0.18 |  | |
| *Drug* | -1.48 | -11.12 – 8.15 | -1.33 | -10.59 – 7.92 |  | |
| *Study* | 1.55 | 0.03 – 3.11 | 1.54 | -3.63 – 6.70 |  | |
| *Age* | 0.46 | 0.27 – 0.67 ^a^ | 0.46 | -0.22 – 1.15 |  | |
| *Sex* | 1.88 | 0.57 – 3.23 ^a^ | 1.85 | -3.15 – 6.85 |  | |
| *CTQ*Time* | 0.01 | -0.03 – 0.04 | 0.01 | -0.03 – 0.04 |  | |
| ***Drug*Time*** | **4.41** | **2.67 – 6.12** | **4.41** | **2.70 – 6.12** |  | |
| *CTQ*Drug* | 0.13 | -0.13 – 0.39 | 0.13 | -0.12 – 0.38 |  | |
| ***CTQ*Drug*Time*** | **-0.06** | **-0.1 – -0.01** | **-0.06** | **-0.10 – -0.01** |  | |
| **More** |  |  |  |  |  | |
| *Time* | 1.1 | -0.46 – 2.68 | 1.1 | -0.49 – 2.69 |  | |
| *CTQ score* | -0.04 | -0.27 – 0.19 | -0.04 | -0.40 – 0.32 |  | |
| *Drug* | -2.44 | -14.36 – 9.17 | -2.57 | -14.64 – 9.50 |  | |
| *Study* | -2.29 | -4.29 – -0.28 | -2.28 | -9.02 – 4.46 |  | |
| *Age* | -0.21 | -0.48 – 0.06 | -0.21 | -1.10 – 0.67 |  | |
| *Sex* | -3.42 | -5.32 – -1.59 | -3.4 | -9.92 – 3.12 |  | |
| *CTQ*Time* | -0.01 | -0.05 – 0.03 | -0.01 | -0.05 – 0.03 |  | |
| ***Drug*Time*** | **6.38** | **4.04 – 8.6** | **6.4** | **4.17 – 8.63** |  | |
| *CTQ*Drug* | 0.12 | -0.2 – 0.45 | 0.13 | -0.20 – 0.45 |  | |
| *CTQ*Drug*Time* | -0.04 | -0.1 – 0.03 | -0.04 | -0.10 – 0.03 |  | |
| Notes. Significant interactions reported by the original analyses are bolded. ^a^ Bootstrapped CI indicate potential difference that is does not concur with original study estimates. | | | | | |  |

| **Table SM8:** Bootstrapped estimates and confidence intervals for buprenorphine models | | | | |
| --- | --- | --- | --- | --- |
|  | **Bootstrapped estimates** | | **Original model** | |
|  | **B: Estimate** | **B: 95% CIs** | **Estimate** | **95% CIs** |
| **Feel** |  |  |  |  |
| *Time* | 12.11 | 4.57 - 19.38 | 12.12 | 4.83 – 19.40 |
| *CTQ score* | 0.37 | -0.28 - 1 | 0.37 | -0.31 – 1.06 |
| *Drug* | 6.1 | -27.89 - 37.59 | 5.92 | -28.26 – 40.09 |
| *Age* | -0.3 | -0.75 - 0.14 | -0.3 | -1.05 – 0.45 |
| *Sex* | 4.19 | -0.19 - 8.62 | 4.17 | -3.41 – 11.74 |
| *CTQ*Time* | -0.19 | -0.39 - 0 | -0.19 | -0.38 – -0.00 |
| *Drug*Time* | -9.73 | -19.24 - 0.6 | -9.67 | -19.97 – 0.64 |
| *CTQ*Drug* | 0.01 | -0.81 - 0.88 | 0.01 | -0.88 – 0.90 |
| *CTQ*Drug*Time* | 0.12 | -0.15 - 0.38 | 0.11 | -0.15 – 0.38 |
| **Like** |  |  |  |  |
| *Time* | 1.38 | -8.02 - 10.79 | 1.47 | -8.01 – 10.96 |
| *CTQ score* | -0.28 | -1.1 - 0.55 | -0.28 | -1.18 – 0.63 |
| *Drug* | -14.77 | -57.17 - 30.37 | -14.43 | -58.92 – 30.07 |
| *Age* | -0.24 | -0.81 - 0.33 | -0.24 | -1.25 – 0.77 |
| *Sex* | -1.52 | -7.29 - 4.41 | -1.56 | -11.78 – 8.66 |
| *CTQ*Time* | 0 | -0.25 - 0.25 | -0.01 | -0.25 – 0.24 |
| *Drug*Time* | 4 | -9.24 - 17.4 | 3.81 | -9.60 – 17.23 |
| *CTQ*Drug* | 0.35 | -0.77 - 1.51 | 0.34 | -0.82 – 1.50 |
| *CTQ*Drug*Time* | -0.09 | -0.44 - 0.26 | -0.08 | -0.43 – 0.26 |
| **Dislike** |  |  |  |  |
| *Time* | 13.43 | 3.89 - 22.3 | 13.5 | 4.43 – 22.58 |
| *CTQ score* | 0.7 | -0.13 - 1.47 | 0.69 | -0.18 – 1.56 |
| *Drug* | 0.73 | -42.5 - 43.66 | 0.35 | -42.21 – 42.91 |
| *Age* | -0.52 | -1.14 - 0.07 | -0.53 | -1.54 – 0.48 |
| *Sex* | 0.93 | -4.72 - 6.68 | 0.81 | -9.38 – 11.00 |
| *CTQ*Time* | -0.14 | -0.38 - 0.11 | -0.15 | -0.38 – 0.09 |
| *Drug*Time* | -11.51 | -24.43 - 0.91 | -11.44 | -24.27 – 1.40 |
| *CTQ*Drug* | 0.25 | -0.86 - 1.38 | 0.26 | -0.85 – 1.37 |
| *CTQ*Drug*Time* | 0.16 | -0.17 - 0.49 | 0.16 | -0.18 – 0.49 |
| **High** |  |  |  |  |
| *Time* | 4.92 | 0.58 - 9.74 | 4.89 | 0.12 – 9.66 |
| *CTQ score* | 0.05 | -0.34 - 0.46 | 0.04 | -0.44 – 0.53 |
| *Drug* | 9.11 | -15.02 - 31.24 | 9.18 | -13.17 – 31.54 |
| *Age* | 0.05 | -0.26 - 0.35 | 0.05 | -0.59 – 0.68 |
| *Sex* | -0.33 | -3.15 - 2.65 | -0.32 | -6.75 – 6.10 |
| *CTQ*Time* | -0.07 | -0.19 - 0.05 | -0.06 | -0.19 – 0.06 |
| *Drug*Time* | -4.97 | -11.99 - 1.8 | -4.93 | -11.67 – 1.81 |
| *CTQ*Drug* | -0.14 | -0.72 - 0.49 | -0.14 | -0.72 – 0.44 |
| *CTQ*Drug*Time* | 0.05 | -0.11 - 0.23 | 0.05 | -0.12 – 0.23 |
| **More** |  |  |  |  |
| *Time* | 6.19 | -1.88 - 13.89 | 6.36 | -1.68 – 14.41 |
| *CTQ score* | 0.1 | -0.6 - 0.8 | 0.11 | -0.71 – 0.93 |
| *Drug* | 8.13 | -28.09 - 45.08 | 8.22 | -29.54 – 45.97 |
| *Age* | -0.2 | -0.72 - 0.32 | -0.2 | -1.27 – 0.88 |
| *Sex* | 4.02 | -1 - 8.78 | 4.01 | -6.82 – 14.83 |
| *CTQ*Time* | -0.12 | -0.33 - 0.09 | -0.12 | -0.33 – 0.09 |
| *Drug*Time* | -2.93 | -13.74 - 8.36 | -2.9 | -14.28 – 8.49 |
| *CTQ*Drug* | -0.32 | -1.26 - 0.64 | -0.32 | -1.30 – 0.66 |
| *CTQ*Drug*Time* | 0.11 | -0.19 - 0.39 | 0.11 | -0.19 – 0.40 |
|  | | | | |

| Table SM9. RI-MLM on the effects of CTQ score on heart rate (bpm) after drug administration. | | | | | | |
| --- | --- | --- | --- | --- | --- | --- |
|  | **Simulant effects on bpm** | | | **Buprenorphine effects on bpm** | | |
| ***Predictors*** | ***Estimates*** | ***95% CIs*** | ***p*** | ***Estimates*** | ***95% CIs*** | ***p*** |
| Time | -0.05 | -0.07 – -0.02 | **<0.001** | -0.07 | -0.12 – -0.03 | **0.002** |
| CTQ score | -0.13 | -0.29 – 0.02 | 0.098 | -0.00 | -0.28 – 0.27 | 0.974 |
| Drug | 0.29 | -3.55 – 4.13 | 0.883 | 3.26 | -5.28 – 11.81 | 0.453 |
| Study | 0.70 | -2.55 – 3.95 | 0.672 |  |  |  |
| Age | -0.18 | -0.59 – 0.24 | 0.411 | 0.18 | -0.34 – 0.70 | 0.503 |
| Sex | 2.94 | -0.25 – 6.13 | 0.071 | -0.96 | -6.19 – 4.28 | 0.719 |
| Time * CTQ score | 0.00 | -0.00 – 0.00 | 0.443 | 0.00 | -0.00 – 0.00 | 0.270 |
| Time * Drug | 0.07 | 0.04 – 0.11 | **<0.001** | 0.01 | -0.05 – 0.08 | 0.692 |
| CTQ score * Drug | -0.02 | -0.12 – 0.09 | 0.725 | -0.10 | -0.32 – 0.12 | 0.373 |
| Time * CTQ score * Drug | -0.00 | -0.00 – 0.00 | 0.819 | -0.00 | -0.00 – 0.00 | 0.816 |
| **Random Effects** | | | | | | |
| σ^2^ | 37.36 | | | 52.73 | | |
| τ_00_ | 41.89 _ID_ | | | 43.42 _ID_ | | |
| ICC | 0.53 | | | 0.45 | | |
| N | 75 _ID_ | | | 34 _ID_ | | |
| Observations | 1072 | | | 340 | | |
| Marginal R^2^ / Conditional R^2^ | 0.190 / 0.618 | | | 0.146 / 0.531 | | |


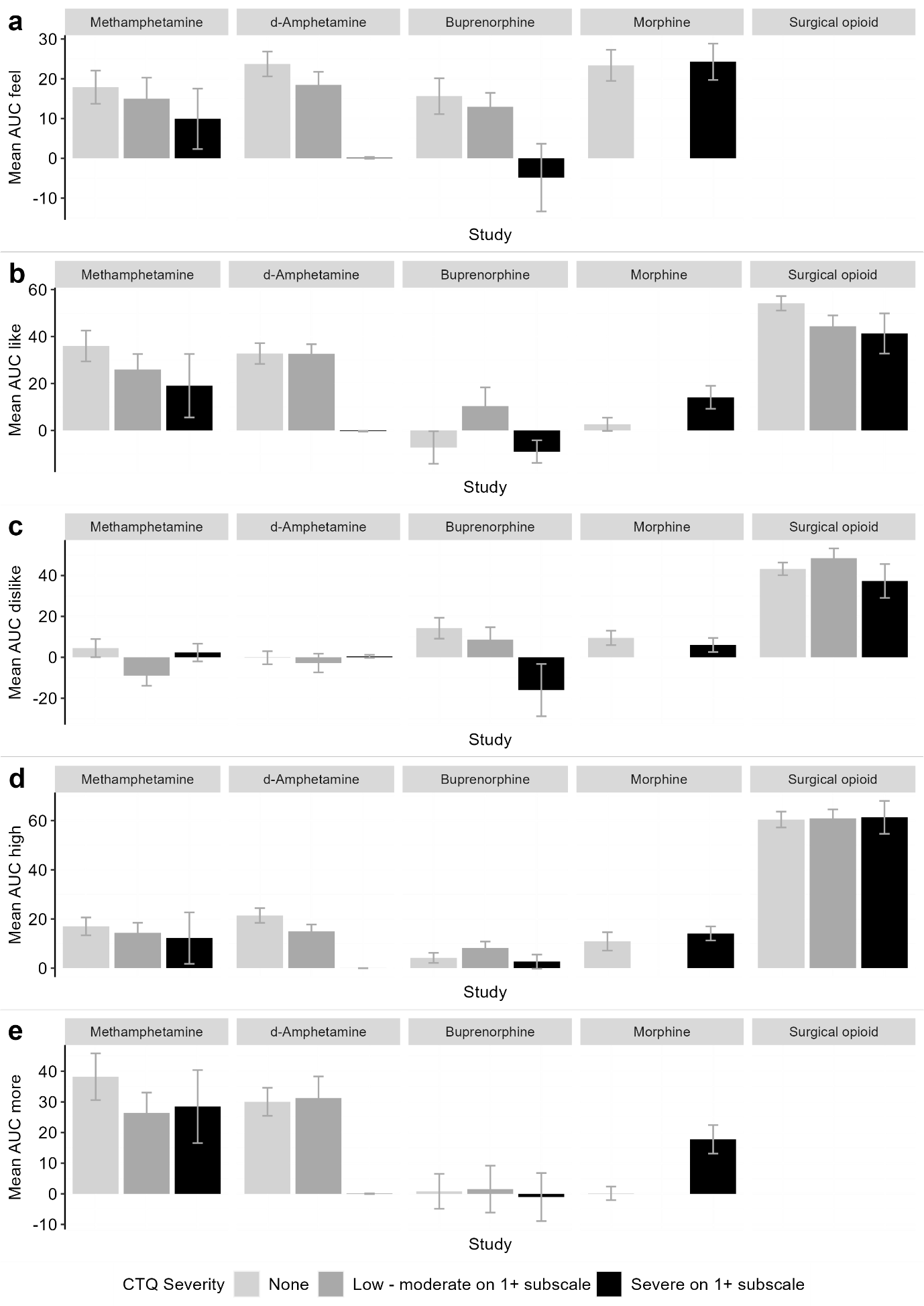


Figure SM10. Group differences in drug responses (average placebo-adjusted AUC) for **(a) feel effects**, **(b) like effects**, **(c) dislike effects**, **(d) feel high**, and **(e) want more** after **methamphetamine, d-amphetamine, buprenorphine, or morphine**, or non-placebo controlled response (0-100) after a **surgical opioid (remifentanil or oxycodone)**.

##


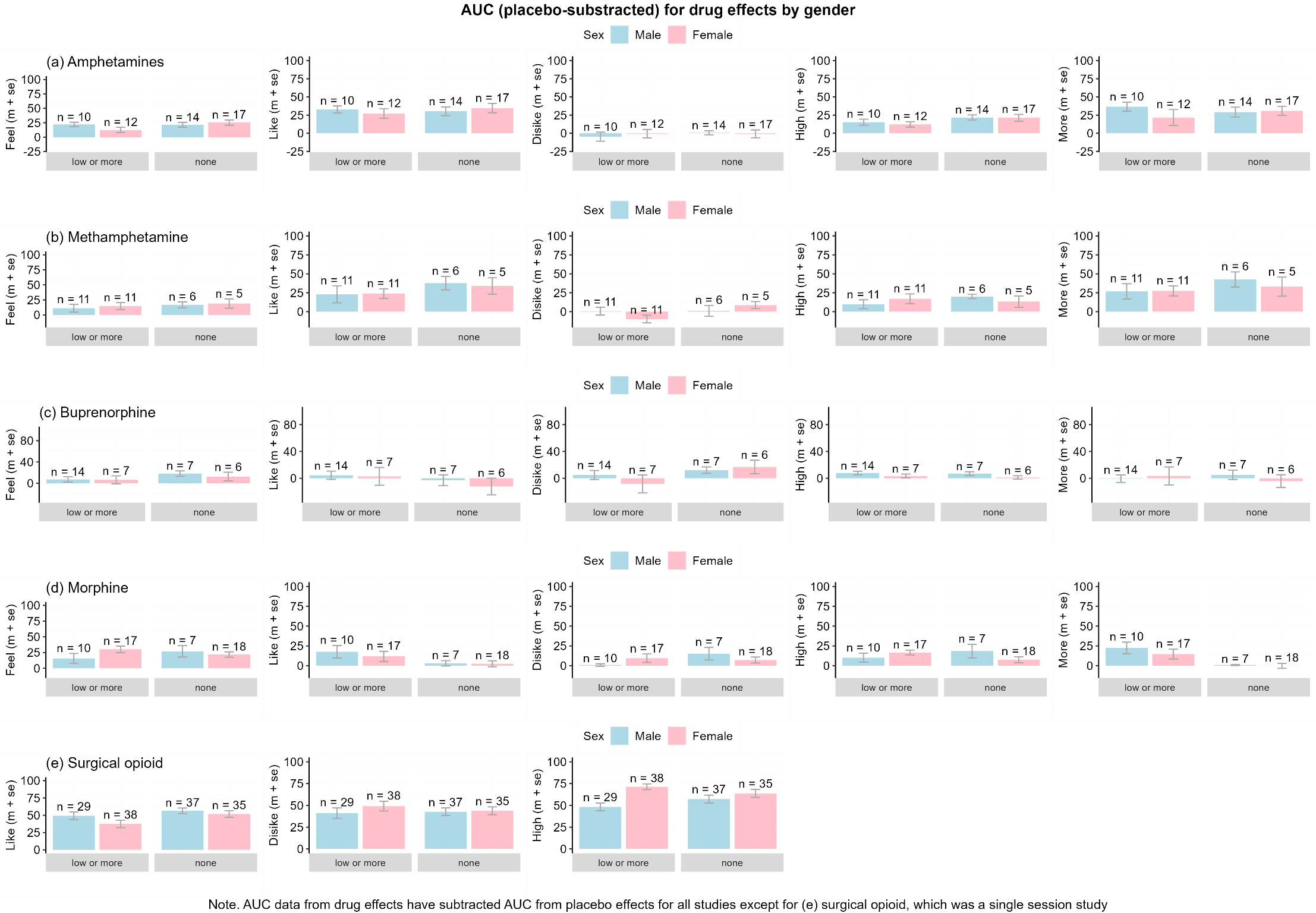

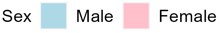


## Figure SM11. Drug responses (AUC) divided by sex and childhood adversity group across all studies
